# Probing elastic anisotropy of human skin in vivo with light using non-contact acoustic micro-tapping OCE and polarization sensitive OCT

**DOI:** 10.1038/s41598-022-07775-3

**Published:** 2022-03-10

**Authors:** Mitchell A. Kirby, Peijun Tang, Hong-Cin Liou, Maju Kuriakose, John J. Pitre, Tam N. Pham, Russell E. Ettinger, Ruikang K. Wang, Matthew O’Donnell, Ivan Pelivanov

**Affiliations:** 1 Department of Bioengineering, University of Washington, Seattle, WA USA; 2 Harborview Medical Center, University of Washington, Seattle, WA USA

**Keywords:** Imaging and sensing, Imaging techniques, Optical imaging, Ultrasound

## Abstract

Skin broadly protects the human body from undesired factors such as ultraviolet radiation and abrasion and helps conserve body temperature and hydration. Skin’s elasticity and its level of anisotropy are key to its aesthetics and function. Currently, however, treatment success is often speculative and subjective, and is rarely based on skin’s elastic properties because there is no fast and accurate non-contact method for imaging of skin’s elasticity. Here we report on a non-contact and non-invasive method to image and characterize skin’s elastic anisotropy. It combines acoustic micro-tapping optical coherence elastography (AμT-OCE) with a nearly incompressible transversely isotropic (NITI) model to quantify skin’s elastic moduli. In addition, skin sites were imaged with polarization sensitive optical coherence tomography (PS-OCT) to help define fiber orientation. Forearm skin areas were investigated in five volunteers. Results clearly demonstrate elastic anisotropy of skin in all subjects. AμT-OCE has distinct advantages over competitive techniques because it provides objective, quantitative characterization of skin’s elasticity without contact, which opens the door for broad translation into clinical use. Finally, we demonstrate that a combination of multiple OCT modalities (structural OCT, OCT angiography, PS-OCT and AμT-OCE) may provide rich information about skin and can be used to characterize scar.

## Introduction

Skin is a complex organ providing a broad spectrum of functions. Its biomechanical properties may depend on environment (such as temperature and humidity), age, gender, body mass index, skin thickness and body site, and any alteration of these properties may indicate disease^1–7^.

Reconstructive surgeries drive the clinical need for non-contact objective measurements of skin elasticity. Skin grafts, including both split thickness and full thickness grafts (STSG or FTSG, respectively), are indispensable techniques to manage complex burn injury, soft tissue injuries, as well as secondary reconstructions. Pathological skin healing is characterized by hypertrophic scarring, whereby thickened and fibrotic tissue cause disfigurement, contractures, and impaired function in survivors. In 2018, there were 17.7 million aesthetic, 1.8 million aesthetic surgical, 15.9 million aesthetic minimally invasive and 5.8 million reconstructive procedures in the United States alone, with a large fraction of interventions involving STSG or FTSG^8^. Skin grafting is one of the oldest and most widely applied reconstructive techniques, finding clinical applications across primary and secondary burn reconstruction, trauma reconstruction, skin oncologic surgery, and many areas of wound care including diabetic foot wounds, venous stasis ulcers, pressure sores, and surgical wounds with delayed healing^9–11^.

FTSG is generally used for aesthetically sensitive parts of the body, such as head and neck regions, and involves harvesting the epidermis with the entire dermis at the subcutaneous/dermal junction from the donor site. FTSG provides improved texture, pliability, elasticity, aesthetics, color match and is more resistant to secondary contracture compared with STSG. FTSG is a sensitive technique requiring multiple steps: donor skin harvest and primary donor site closure, recipient site preparation, graft placement and securement, graft immobilization, and long-term postoperative care and monitoring as the graft undergoes the standard phases of wound healing including inflammatory (4–6 days), proliferative (up to 3 months) and remodeling (up to a year or year and a half) phases^10,11^.

A skin graft’s functional and mechanical properties must be matched with surrounding recipient tissue to restore both form and function and minimize scar. This is especially true for FTSG surgery undertaken in the face or neck. Recent studies suggest that tissue elasticity is a critically important parameter driving reconstructive success^12,13^. Indeed, collagen fibers mainly determine Young’s modulus and elastic anisotropy (Langer’s lines). Although medical providers have long recognized that linear surgical incisions placed along Langer’s lines heal with less tension and scarring, it remains unclear how to best leverage elastic anisotropy when replacing larger defects through skin grafting. Thus, STSG and FTSG’s functional and aesthetic results will be severely limited without matching the elasticity of adjacent recipient tissue^14–16^. Currently, there are no non-contact methods to monitor skin elastic properties, especially its anisotropy.

Recently, optical coherence elastography (OCE) was proposed to remove the last drawback in shear wave elastography, i.e., to make this method fully non-contact^17^. Although static and vibrational OCE still require tissue contact, dynamic OCE does not require it at all. Indeed, in 2016, air-coupled ultrasound was proposed to launch sub-mm wavelength propagating mechanical waves in tissue via reflection-based acoustic radiation force^18^. This acoustic micro-tapping method (AμT) was combined with phase-sensitive OCT to create a fully non-contact method (AμT-OCE) of elasticity imaging in tissues^19^.

Despite the remarkable success in imaging propagating mechanical waves in vivo in cornea and skin with AμT-OCE, interpreting results and converting wavefields into elastic moduli has been unclear for some time. Indeed, both cornea and skin are anisotropic, bounded, layered media. Wave propagation in such media is complicated and reconstructing elastic moduli using surface-propagating mechanical waves is not trivial. As such, an appropriate mechanical model is needed to solve this problem. Recently, we developed a model of a nearly incompressible transversely isotropic (NITI) medium^20^, opening the way for quantitative evaluation of anisotropic elastic properties in biological tissues. We used the NITI model to quantify corneal elasticity and justified it by direct comparison with mechanical tests^21^.

Numerous mechanical tests have evaluated the elastic behavior of skin, including indentation, torsion, tension, and suction^22–26^; corresponding tools were commercialized (Dermaflex^27^ and Cutometer^28^). All such methods have similar disadvantages. Most use simple linear stress–strain relationships, depend on tip geometry, ignore skin’s multilayered structure and anisotropy, do not account for skin thickness, and require contact. Although they are occasionally used in dermatology, contact tests are not currently used to monitor plastic and burn surgeries, and skin graft outcomes, because physical contact produces considerable patient discomfort and measurements are not sufficiently objective or reproducible to guide clinical decision making^27^.

Young’s moduli of skin reported in the literature demonstrate dramatic variations depending on the measurement technique^29^. The range of moduli reported from indentation tests is usually from Pa to tens of kPa^30–32^, with great variability depending on the probe size used^33^; suction tests report values about hundreds of kPa^34–36^; torsion tests find Young’s moduli on the order of MPa^37,38^; and tensile measurements sometimes report values of hundreds of MPa for the Young’s modulus in skin^39–41^. Thus, six orders of magnitude difference in Young’s modulus can be found in the literature.

An additional complication in characterizing skin’s elasticity is its non-linearity, i.e., elastic moduli depend on the tensile or deformation applied during measurements. The larger the deformation applied, the larger the Young’s modulus usually measured^29,33^. This is why low-deformation methods are of critical importance to characterize skin under normal, physiologic conditions that do not modify the object during measurement.

Current tools to evaluate scar longitudinally include the Vancouver Scar Scale (VSS) and the Patient and Observer Scar Assessment Scale (POSAS)^42^. They include assessment of parameters such as pliability, firmness, color, perfusion, thickness, and 3-dimensional topography. While validated, they are both subject to observation bias and cannot quantitatively evaluate changes within scar tissue.

Acoustic elastography is a low-deformation method using propagating mechanical waves to probe tissue elasticity. Their excitation and detection can be done in different ways. Originally, a mechanical vibrator in direct contact with tissue produced transient displacements^43^; later, the vibrator was replaced by acoustic radiation force generated by a focused ultrasound (US) beam^44,45^. Note that shear wave elastography has evolved into an indispensable clinical tool, especially for the liver and breast^46,47^. However, shear wave elastography has not significantly impacted clinical applications in skin even though multiple studies have been published. The main reason is that US coupling material must be applied to the skin surface, representing a significant site of contact; in addition, US coupling changes skin’s hydration and is not ideal for burns and grafts.

The mechanical model is critically important to reconstruct material mechanical moduli from experimental data (including methods using wave propagation). Reconstructed moduli can then be used in a computational model to predict tissue deformation based on applied loads. Soft biological tissues are nearly incompressible^48^. If the medium is also isotropic, there is only one parameter (shear modulus $\mu$) defining both shear and tensile deformations; the Poisson’s ratio approaches 0.5 and the Young’s modulus is $E=3\mu$. Thus, $\mu$ is the sole parameter defining the deformation of a linear, isotropic, incompressible elastic material.

Unfortunately, skin is not isotropic^49,50^. The simplest model accounting for skin’s anisotropy is transversal isotropy (TI) with a symmetry axis defined by Langer’s lines, i.e., fiber orientation. For a nearly incompressible transversely isotropic (NITI) material (see Results and Supplementary Notes), an additional shear modulus $G$ and parameter $\delta$ are needed along with $\mu$ to describe shear and tensile deformations in different directions relative to Langer’s lines.

In this study, we further develop the NITI model^20^ (recently introduced for cornea) for skin-type material anisotropy, considering it as a locally transverse isotropic material with a symmetry axis defined by Langer’s lines. We used an AμT-OCE system to image propagating waves along skin’s surface in human forearm in vivo in five healthy volunteers. For each subject, we varied the Rayleigh (surface) wave propagation direction relative to Langer’s lines and reconstructed all three independent shear moduli. AμT-OCE measurements can also define the material symmetry axis, but it is desirable to measure the fiber orientation in skin independently to improve reconstruction accuracy.

An independent measurement of skin’s optic axis (or birefringence axis) was performed with polarization-sensitive OCT (PS-OCT)^51^. Both AμT-OCE and PS-OCT measurements revealed very similar orientation of the symmetry axis in the epidermis for all measured subjects. To our knowledge, non-contact quantitative evaluation of skin’s elasticity and its anisotropy, with the symmetry axis confirmed by independent PS-OCT measurements, has not been demonstrated before. In addition, we present pilot results on imaging scar in vivo and show that rich information can be obtained non-invasively with four different OCT modalities (structural OCT, OCT angiography, OCE and PS-OCT). Combining measurements from these modalities can provide comprehensive quantitative characterization of skin and pave the way for large-scale clinical studies in the future.

## Results

### Nearly incompressible transverse isotropy (NITI) of skin’s elasticity

Dynamic OCE uses three principal steps: (1) excite propagating mechanical waves (surface waves in our case), (2) track propagating mechanical waves with phase sensitive OCT and (3) reconstruct tissue moduli from surface wavefields using a mechanical model. Even if the first two steps are done properly, an inappropriate model will produce an incorrect reconstruction of medium mechanical properties.

An example of the importance of using a correct mechanical model can be found in OCE applied to the cornea, where the literature reported orders of magnitude mismatch in corneal elasticity obtained with OCE compared to that measured with mechanical tensile tests. Originally, the cornea was incorrectly considered an isotropic material. With the recent introduction of the appropriate NITI model, reconstructed moduli closely match those measured with conventional destructive mechanical tests. Indeed, the random in-plane orientation of corneal lamellae supports the assumption of in-plane isotropy, but very different mechanical behavior out-of-plane. Thus, at least two independent moduli must be considered for cornea. Further details on how corneal anisotropy influences wave behavior and affects moduli reconstruction can be found in Ref.^20^.

We believe a similar approach must also be used for skin. In other words, skin’s mechanical anisotropy must be taken into account. Indeed, Langer’s lines define the primary orientation of fibers in skin. Although a fraction of collagen fibers may be oriented perpendicular to Langer’s lines and some by 45°, the majority are oriented along this direction^52,53^. It means that, macroscopically, skin should behave as a NITI medium with a symmetry axis (Z-axis) associated with Langer’s lines.

We start our description of a model for skin’s elasticity with a general form of the elastic modulus matrix for a transversely isotropic (TI) material:

1 $$C=\left[\begin{array}{cccccc}{C}_{11}& {C}_{12}& {C}_{13}& & & \\ {C}_{12}& {C}_{11}& {C}_{13}& & & \\ {C}_{13}& {C}_{13}& {C}_{33}& & & \\ & & & {C}_{44}& & \\ & & & & {C}_{44}& \\ & & & & & {C}_{66}\end{array}\right],$$

where ${C}_{12}={C}_{11}-2{C}_{66}$ due to symmetry conditions. Since one principal plane in the TI material is isotropic, we use the conventional notation of an isotropic material with a few TI modifications^48^:

2 $$C=\left[\begin{array}{cccccc}\lambda +2\mu & \lambda & \lambda +{Q}_{1}& & & \\ \lambda & \lambda +2\mu & \lambda +{Q}_{1}& & & \\ \lambda +{Q}_{1}& \lambda +{Q}_{1}& \lambda +2\mu +{Q}_{2}& & & \\ & & & G& & \\ & & & & G& \\ & & & & & \mu \end{array}\right],$$

where $\lambda$ and $\mu$ are the conventional Lamé constants. An additional modulus $G$ shows that shear deformation can be different if shear stress is applied along the symmetry axis $Z$ rather than across it. Modulus $G$ can be very different from $\mu$ in soft tissue. In cornea, for instance, $\mu /G$ can be more than a hundred^20^.

Tensile deformations include the additional parameters ${Q}_{1}$ and ${Q}_{2}$. Soft biological tissue is nearly incompressible so that shear moduli are many orders of magnitude smaller than longitudinal ones and therefore

3 $${Q}_{1}, {Q}_{2}\sim \mu \ll \lambda .$$

Nevertheless, these small parameters are important in characterizing Young’s moduli along, ${E}_{L}$, and across, ${E}_{T}$, fibers, i.e., assuming that tensional deformation is different along the fiber direction compared to that across it.

In the isotropic, incompressible limit, $\mu =G$, Young’s modulus ${E}_{L}={E}_{T}=3\mu$, and Poisson’s ratio $\nu =1/2$, i.e., both tensile and shear deformations can be characterized by a single parameter $\mu$.

In Supplementary Note 1 we show how Young’s moduli ${E}_{L}$ and ${E}_{T}$, and Poisson’s ratios are defined through ${Q}_{1}, {Q}_{2}$ and $\mu$ and how different they can be from $3\mu$ for skin-type NITI material. In summary, these important parameters can be written as (see Supplementary Note 1):

4 $$\begin{array}{c}{E}_{T}=3\mu +\mu \left[\frac{\delta }{4\mu +\delta }\right] ,\\ {E}_{L}=3\mu +\delta , \\ { \nu }_{TT}=\frac{1}{2}\left[1+\frac{\delta }{4\mu +\delta }\right]=1-\frac{1}{2}\frac{{E}_{T}}{{E}_{L}} ,\\ { \nu }_{TL}=\frac{1}{2}\left[1-\frac{\delta }{4\mu +\delta }\right]=\frac{1}{2}\frac{{E}_{T}}{{E}_{L}},\\ {\nu }_{LT}=\frac{1}{2} ,\end{array}$$

A few important observations can be drawn from these expressions: (1) compared to the isotropic case, there are two parameters: $\mu$ and an additional parameter $\delta ={Q}_{2}-2{Q}_{1}$ that define the Young’s moduli and Poisson’s ratios. (2) All Young’s moduli and Poisson’s ratios do not depend on the shear modulus $G$. (3) The fact that ${\nu }_{LT}$ is equal to 1/2 means that the deformation will be distributed equally in the isotropy plane when the stress is applied along the symmetry axis. (4) However, when the stress is applied perpendicular to the fiber direction, the deformation will be distributed unequally along and perpendicular to the fibers, but the sum of them is equal to unity:

5 $${\nu }_{TT}+{\nu }_{TL}=1.$$

(v) The fact that ${\nu }_{TT}>0$ imposes limitations on the relationship between ${E}_{L}$ and ${E}_{T}$ and their absolute values:

6 $$\begin{array}{c}{E}_{L}>{E}_{T}/2\\ {E}_{L}>\mu \\ {E}_{T}>2\mu \end{array}.$$

Thus, there are three parameters primarily describing simple tensile and shear mechanical properties in the skin-type NITI material: $\mu$, ${\delta =Q}_{2}-2{Q}_{1}$, and $G$.

Supplementary Fig. 1 shows how Young’s moduli can change with $\delta$. Clearly, ${E}_{T}$ has a very narrow range, with a lower limit of $2\mu$ and upper limit of $4\mu$ as $\delta \to \infty$. ${E}_{L}$ is a linear function of $\delta$.

Since all Poisson’s ratios should be greater than 0 for normal materials, this further narrows the range of ${E}_{T}$ to

7 $${3\mu <E}_{T}<4\mu .$$

In practice, ${E}_{L}$ is usually larger than ${E}_{T}$ (Young’s modulus along fibers is larger than that across), and the ratio of moduli is also usually limited by a factor ${E}_{L}/{E}_{T}<\sim 2$^49,50^, i.e., ${E}_{L}<\sim 7\mu$.

Note that skin is a multi-layered medium composed of epidermis, dermis, and hypodermis (subcutaneous tissue). The outermost epidermal layer acts as a barrier but does not greatly affect skin’s elastic behavior because it is very thin (~ 150 μm). The deepest layer, hypodermis, connects skin to muscle and acts mostly like a mechanical damping layer combined with thermal insulation. Because subcutaneous tissue contains less fiber and more fat, its elasticity is much smaller than that of dermis. The central layer, dermis, consists of elastin and collagen fibers that account for about 90% of skin’s weight, and defines most of the mechanical elasticity and anisotropy in skin^54^. Thus, dermis is of primary interest.

Uniaxial tensile and inflation tests yield Young’s modulus estimates related to $\mu$ and $\delta$. However, shear torsional tests depend only on *G*. This decoupling of normal and shear deformations helps explain the discrepancy between tensile/inflation test modulus estimates.

### Wave propagation in a NITI medium

As discussed above, we assume that skin’s elastic properties are mostly driven by the dermis, which can be described as a NITI material. A NITI medium supports three bulk waves—quasi-longitudinal, quasi-shear, and shear. Soft tissues are nearly incompressible ($\lambda$ ≫ $\mu$), implying that the quasi-longitudinal wave speed is nearly constant along all directions. However, quasi-shear and shear wave speeds do vary with angle and depend on both $G/\mu$ and $\delta /\mu$ (see Supplementary Note 2)^55,56^. Thus, the measurement of angle-dependent anisotropy in shear and quasi-shear wave propagation can be used for elastic moduli reconstruction in skin.

Directly monitoring bulk shear wave propagation in the dermis is problematic. Indeed, the thickness of the dermis varies between 0.3 and 3 mm, and shear-wave propagation could be guided. Although multiple studies were performed using conventional shear wave elastography in skin^57–59^, only a few discussed anisotropy^60^ and none (to our knowledge) considered the influence of boundaries. Ignoring these factors can lead to serious errors in moduli reconstruction due to strong frequency dispersion. In addition, accounting for an irregular and not well-defined transition between dermis and hypodermis is not a simple task.

An alternate approach measures the anisotropy of surface waves (as it is done in dynamic OCE), i.e., waves propagating along the skin surface in different directions relative to Langer’s lines. In Supplementary Note 3, we showed that a solution to the angular dependence of Rayleigh (surface) wave speed can be obtained using the Stroh formalism^61,62^.

**Figure 1:**
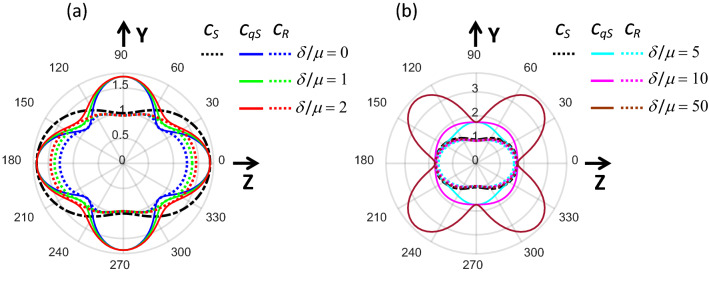
Anisotropy of phase velocity for bulk shear (black dashed line), quasi-shear (solid lines) and surface Rayleigh (dotted lines) waves in a fast-axis NITI medium. (**a**) $\delta /\mu <2$, (**b**) $\delta /\mu >5$. In both panels $G/\mu =3$.

Figure 1 shows the Raleigh wave velocity as a function of propagation angle in the YZ plane using the coordinate system defined in Supplementary Note 2 at different parameters $G/\mu$ and $\delta /\mu$; the velocities of shear ${c}_{S}$ and quasi-shear ${c}_{qS}$ waves are also shown for reference. Note the fundamental difference between waves propagating through the volume of a NITI material and over its surface.

When the Rayleigh wave propagates perpendicular to the symmetry axis over the surface of a fast-axis NITI medium (i.e., for the case of $\delta /\mu >0$ as defined in Supplementary Note 1), its speed (${c}_{R}\left(\vartheta \right)$) is solely defined by $\mu$ and does not depend on *G* and $\delta$, i.e., it equals that for an isotropic material:

8 $${{c}_{R}\left(\vartheta =90^\circ \right)=0.9553c}_{S}\left(\vartheta =90^\circ \right)=0.9553\sqrt{\mu /\rho },$$

However, when the Rayleigh wave propagates along the fibers, its speed is a function of all parameters *G,* $\mu$ and $\delta$ and cannot be represented in a simple form. The solution of the secular equation (S27) (see Supplementary Note 3) can be done numerically, or using a combination of parameters in Eq. (S27), as shown by Abramowitz in Ref.^63^.

An interesting observation is that when $\delta$ is smaller than $G$ (see Fig. 1a), at $\vartheta =45^\circ$, the Rayleigh wave speed

9 $${{c}_{R}\left(\vartheta =45^\circ \right)\approx c}_{qS}\left(\vartheta =45^\circ \right)=\sqrt{\frac{\mu +\frac{\delta }{4}}{\rho }} ,$$

does not depend on $G$.

Although the quasi-shear wave speed is not limited by $\sqrt{G/\rho }$ and can change broadly with $\delta$, the Rayleigh wave speed in the NITI medium cannot exceed $\sqrt{G/\rho }$. This limit is reached when $\delta /\mu \to \infty$ (see Fig. 1b). This fact limits the sensitivity of using Rayleigh waves to invert elastic moduli when $\delta /\mu >\sim 10$, where the Rayleigh wave speed approaches that of the shear wave (Fig. 1b):

10 $${{c}_{R}\left(\vartheta ,\frac{\delta }{\mu }>\sim 10 \right)\approx c}_{S}\left(\vartheta \right)=\sqrt{\frac{G{\mathrm{cos}}^{2}\vartheta +\mu {\mathrm{sin}}^{2}\vartheta }{\rho }}.$$

Fortunately, anisotropy is not so strong for human skin^49,50^. Because the Rayleigh wave speed is a unique function of parameters $\mu$, $G$ and $\delta$, it can be used to reconstruct elastic properties in skin, i.e., skin’s Young’s moduli and Poisson’s ratios outlined in Eq. (4). Additional details on Rayleigh wave anisotropy can be found in Supplementary Note 3.

There may be complications, similar to that for bulk waves, because of limited dermal thickness and boundaries. This is a complex problem, in general, but it can be simplified by considering all scales carefully. Indeed, a thin, compared to the mechanical wavelength, bounding layer barely affects the surface wave speed (see Supplementary Note 4). Subcutaneous tissue should produce guided waves with the dispersion determined by both dermis and hypodermis properties (see Supplementary Note 5).

Surprisingly, surface wave guidance was not observed in our experiments. In addition, we performed auxiliary experiments in chicken drumsticks where we explored surface wave propagation for two situations. First, OCE experiments were performed in whole chicken drumsticks (see Supplementary Note 6) and no guided wave behavior was observed. Then, skin was removed from muscle and positioned on top of water. In this case, clear guided wave behavior was recorded. Reconstruction of shear wave speed from the dispersion analysis of guided waves in excised skin yielded a similar value to that obtained from group velocity analysis for the whole chicken drumstick.

We assume that these results can be explained by two factors: (1) small differences in elastic properties between skin layers and (2) an irregular transition between layers. Both factors make reflections from the skin/subcutaneous tissue interface inefficient, which strongly reduces guided behavior. Thus, mechanical wave propagation along the skin surface can be considered propagation along the surface of a bulk NITI medium.

### Elastic anisotropy of skin in human forearm: in vivo measurement with AμT-OCE

**Figure 2:**
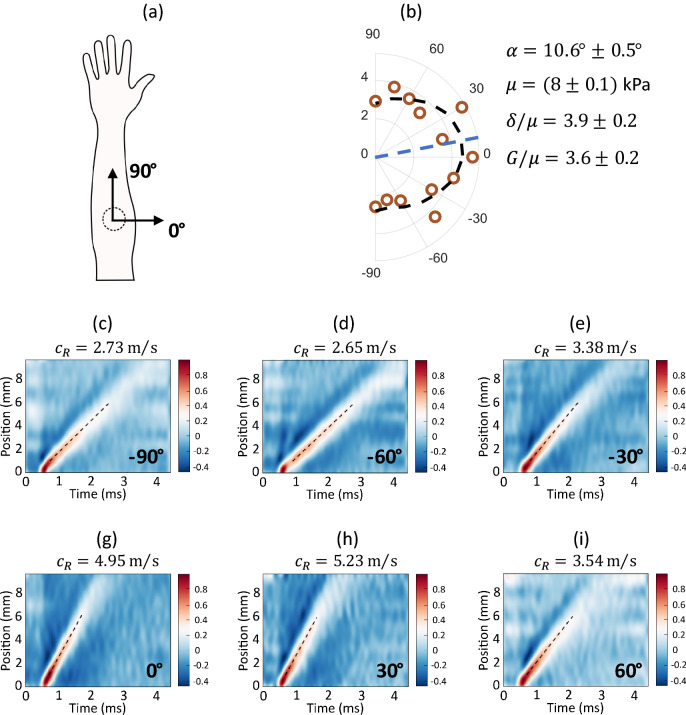
An example of in-plane anisotropy of Rayleigh wave propagation in skin (forearm body area, Subject #4). (**a**) Schematic of the measurement site. Imaging of propagating mechanical waves was performed with AμT-OCE in different propagation directions in human forearm in vivo. Zero propagation angle $\vartheta$ corresponds to the direction perpendicular to the axial forearm direction, which corresponds to the direction of traditional Langer’s lines. (**b**) Measured anisotropy of Rayleigh wave speed in Subject #4 (dots) and the best fit to the analytic solution derived from the NITI model (see Supplementary Note 3). The defined orientation of the mechanical symmetry axis (Langer’s lines) $\alpha =(10.6\pm 0.5)^\circ$, and the shear modulus $\mu$ and anisotropy factors correspond to the values at minimum fit error. (**c**–**i**) Measured wavefields of Rayleigh waves at different in-plane propagation angles.

A spectral-domain OCT system with a 46.5 kHz A-line rate operating in MB-mode^64^ was used to track propagating mechanical waves over the skin surface in healthy human volunteers’ forearms in vivo. A detailed description of the system can be found in our previous studies^18–20,64,65^. Briefly, a cylindrically focused 1 MHz air-coupled ultrasound transducer (AμT) provided a spatially and temporally sharp push to the skin surface in the investigated body site, generating mechanical waves with a bandwidth up to 4 kHz (see “Methods” section). For each human subject, measurements were performed at different propagation angles relative to a chosen coordinate system. Because Langer’s lines in the forearm are traditionally described as orthogonal to the axial forearm direction, the coordinate system was chosen as represented in Fig. 2a.

The anisotropy of surface wave group velocity measured with AμT-OCE is presented in Fig. 2b with dots. Individual wavefields (collection of signal profiles recorded at different distances from the AμT source) are presented in Fig. 2c–h for different propagation directions over the skin surface. There were 13 datasets for each human subject corresponding to a range of propagation directions between − 90° and 90° with a step of 15°.

There are a few important observations from Fig. 2c–h. First, recorded wavefields do not look dispersive as we observed previously in cornea^20^ or in chicken skin placed on water (see Supplementary Note 6). It means that subcutaneous tissue has little influence on surface-propagating mechanical waves and the wave speed can be characterized with the group velocity. For every propagating direction, we fit wavefields with a linear function; fitting results are shown on the top of each panel in Fig. 2c–h and summarized in Fig. 2b for all propagation directions. Second, the speed of surface waves is angle-dependent, which confirms that skin is elastically anisotropic.

**Figure 3:**
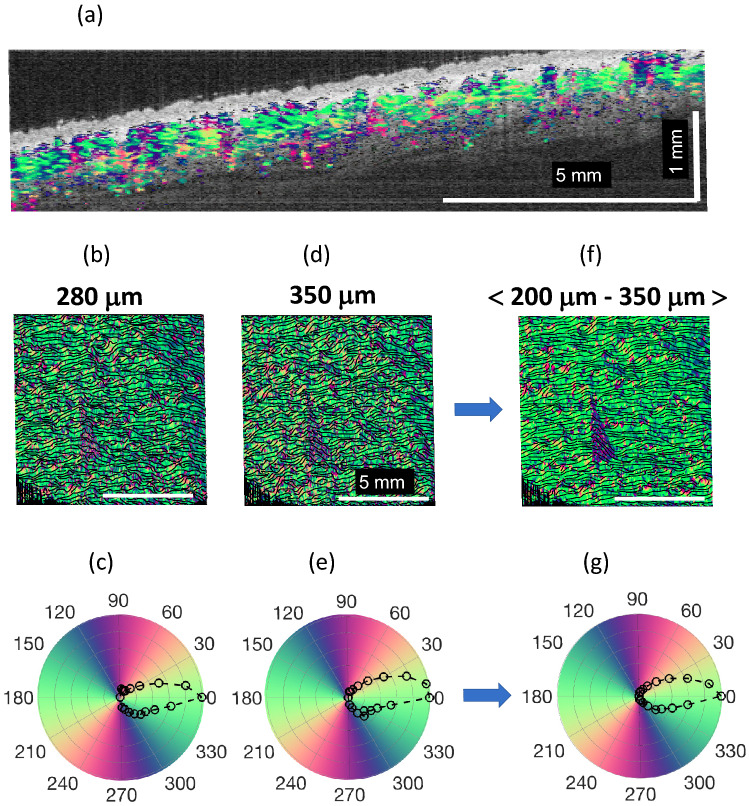
Optical polarization maps in skin (forearm body area, Subject #4). PS-OCT measurements were performed at the same body sites to compare with AμT-OCE. (**a**) Cross-sectional OCT image with the depth-resolved optic axis orientation measurement superimposed. (**b**), (**d**), (**f**) Color-encoded en-face slices (i.e., C-Scan) of the apparent optic axis at different depths in skin (depths are shown at the top of panels), and (**c**), (**e**), (**g**) present in-plane optic axis patterns averaged over skin maps for the same depths. Last panels (**f**) and (**g**) are obtained by averaging optic axis orientations over the entire depth in dermis. A piece of triangular black tape was used to align PS-OCT relative to AμT-OCE measurements.

Fitting Rayleigh wave anisotropy with the theoretical function (see Fig. 1) can be used to extract elastic moduli $\mu$, $G$ and $\delta$, which can be used to calculate the tensile anisotropy (${E}_{L}/{E}_{T}$) and shear anisotropy ($G/\mu$). We fit OCE data using four parameters, with the initial guess of the skin’s actual fiber orientation $\alpha$ relative to the classic orientation of Langer’s line in the forearm (see Fig. 3a). To determine actual inaccuracies in estimated fit parameters, a ‘Leave-one-out cross-validation’ method was used^66^. A total of N − 1 data points were used to fit the data N times, and the average over *N* values produced estimates of the mean for all fit parameters.

For the subject (Subject #4) presented in Fig. 2b, the orientation of the mechanical axis, i.e. Langer’s lines, was determined to be $\alpha =10.6^\circ \pm 0.5^\circ$. This value is not zero; that is, Langer’s lines are not exactly perpendicular to the axial forearm direction. Values for $\alpha$*,* $\mu$, $\delta /\mu$ and $G/\mu$ are shown in the right bottom corner of Fig. 2b. Clearly, skin anisotropy in the forearm is quite strong for Subject #4. It means that tissue deformation across Langer’s lines should be about 2 times greater than along them (${E}_{L}/{E}_{T}=1.98$) for the same applied one-dimensional load, which is very important to know when planning graft placement, orientation and predicting secondary contractions from skin graft surgeries.

### Fiber orientation in skin: measurement with PS-OCT

To determine whether measured mechanical anisotropy in skin correlates with its constituent structure, the skin of each volunteer was also probed at the same sites with PS-OCT. We assume that collagen fibers are the dominant birefringent scattering component in skin. Assuming that the fibers define mechanical anisotropy, collagen fibers should also be anisotropic in orientation. Fiber orientation and mechanical symmetry axes are not always well aligned, but alignment here provides strong evidence that OCE data processing with the NITI model correctly defines mechanical symmetry.

Polarization-sensitive optical coherence tomography (PS-OCT)^51,67–70^, an extension of conventional optical coherence tomography (OCT), can characterize cross-sectional birefringent biological structures non-invasively and can be used to determine the anisotropic orientation of collagen fibers embedded within skin. In this study, the same forearm region scanned via OCE was imaged using the PS-OCT system described in Refs.^69,70^ (see also details in the “Methods” section). This system provided depth-resolved axis orientation mapping of collagen fibers in the skin up to a depth of ~ 500 μm.

Figure 3 presents collagen fiber directionality based on the depth-resolved apparent optic axis in Subject #4. Non-birefringent components were removed using a color filter before computing the axis orientation^68^. The relative optic axis was mapped to a color-wheel where 0° was defined to align with the coordinate system in OCE. It is interesting (see Fig. 3a) that there is little birefringence in a thin layer beneath the skin surface (up to a 100 μm depth) compared to deeper skin tissue, suggesting poor collagen organization in the superficial layer. This thin layer corresponds to the epidermis, which can be seen in the cross-sectional structure image. It is uncolored at most points of the image (Fig. 3a), indicating that the epidermal layer does not change the polarization state of the probing light beam. Hence, the epidermis is optically, minimally birefringent due to very little collagen fiber content in it. Note that the epidermal layer is not captured by AμT-OCE measurements because it is very thin compared to the mechanical wavelength.

Although fibers are oriented differently in the dermis (see Fig. 3a), the preferred optic axis orientation between ~ 100 μm and 350 μm depth is quite clear (see Fig. 3b–e for individual layers, and the accumulated scattering anisotropy is seen in Fig. 3f, g). The accumulated (averaged) optic axis orientation in the dermis can be compared with the orientation of the mechanical axis obtained with AμT-OCE. Unfortunately, the sensitivity of the current PS-OCT system is not enough to see if there is a preferred fiber orientation in subcutaneous tissue.

### Summary of anisotropy in skin of five healthy human subjects

**Figure 4:**
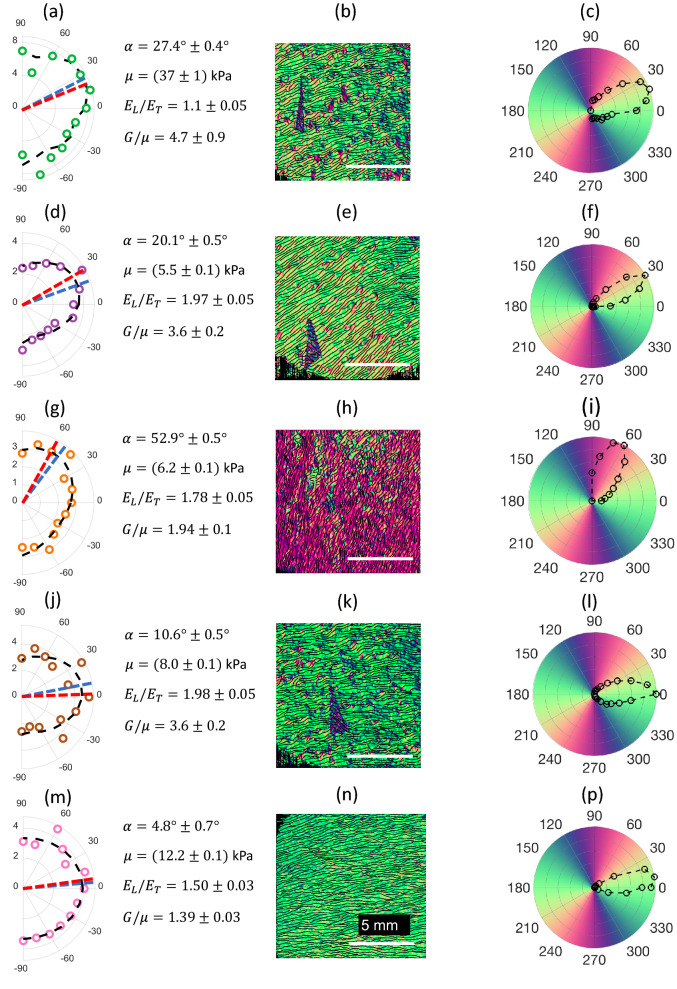
Comparison of AμT-OCE with PS-OCT measurements for all 5 human subjects. Left column (panels (**a**), (**d**), (**g**), (**j**) and (**m**)) present AμT-OCE measurements of mechanical anisotropy in skin in human forearm body sites in vivo. Central column (panels (**b**), (**e**), (**h**), (**k**) and (**n**)) show optic axis maps integrated over the dermis obtained with PS-OCT, and the right column (panels (**c**), (**f**), (**i**), (**l**) and (**p**)) shows directivity patterns of optical polarization in dermis integrated over the dermis thickness at the same body sites. Both methods reveal a similar symmetry direction for all human subjects. Blue and red dashed lines in the left column correspond to the skin’s symmetry axis determined from AμT-OCE and PS-OCT, respectively. A triangular piece of black tape helped align PS-OCT coordinates relative to AμT-OCE measurements.

Measurements in skin in the forearm of five healthy human subjects were performed with both AμT-OCE and PS-OCT methods. Data obtained for every subject were processed as described in the previous 2 sections. Results are summarized in Fig. 4.

It is interesting, and encouraging, that the symmetry axes derived from optical and mechanical measurements match very closely for all investigated subjects. In general, optical symmetry is not always equal to the mechanical symmetry of a material. For human forearm skin, however, it appears that optical and mechanical symmetry axes coincide.

As reported in the literature on Langer’s lines in the forearm, the direction should be nearly orthogonal to the axial forearm direction ($\alpha$ should be around zero). In our measurements, the direction of Langer’s lines was close to orthogonal in only 2 subjects (Subjects #4 and #5); in two subjects, the inclination $\alpha$ was about 30°; and in one case $\alpha$ was about 60°.

An interesting observation can be found from the results shown in Fig. 4. For four of five subjects (subjects #2–#5), a higher value of shear anisotropy $G/\mu$ corresponds to higher tensile anisotropy ${E}_{L}/{E}_{T}$. Both ratios depend on the relative density of fibers and their alignment in measured sites and may be functions of multiple physiological parameters. This observation is not statistically significant but is the subject of a future study that will be performed in a much larger population with subgroups of different body sites, age, sex, body mass index and other parameters.

The fact that all elastic moduli ($G$, $\mu$ and $\delta$) are much larger for subject #1 compared to other subjects strongly imply that skin’s elastic properties can vary greatly from person to person. This suggests that personalized treatment plans are more likely required for both aesthetic and reconstructive skin procedures.

### Imaging of scar in human wrist in vivo with multiple OCT modalities: preliminary results

In recent decades, several optical diagnostic methods have been proposed to image skin and evaluate scar. OCT is one of the most promising methods to provide diverse information on skin constituents. Indeed, signal intensity in structural OCT images is driven by tissue scattering properties, which are related to collagen density. OCT angiography (OCTa) can image microvasculature. PS-OCT images skin’s optical anisotropy by measuring optic (birefringence) axis orientation, and OCE can image skin’s elastic properties. Although individual OCT modalities were used to compare normal skin structure and scar before, we do not know of any studies that have demonstrated all four OCT modalities to characterize the same scar tissue. Here we present our pilot results on imaging scarred skin with different OCT modalities and show that the information obtained can be very rich, diverse and useful for clinicians to evaluate skin’s state and function.

**Figure 5:**
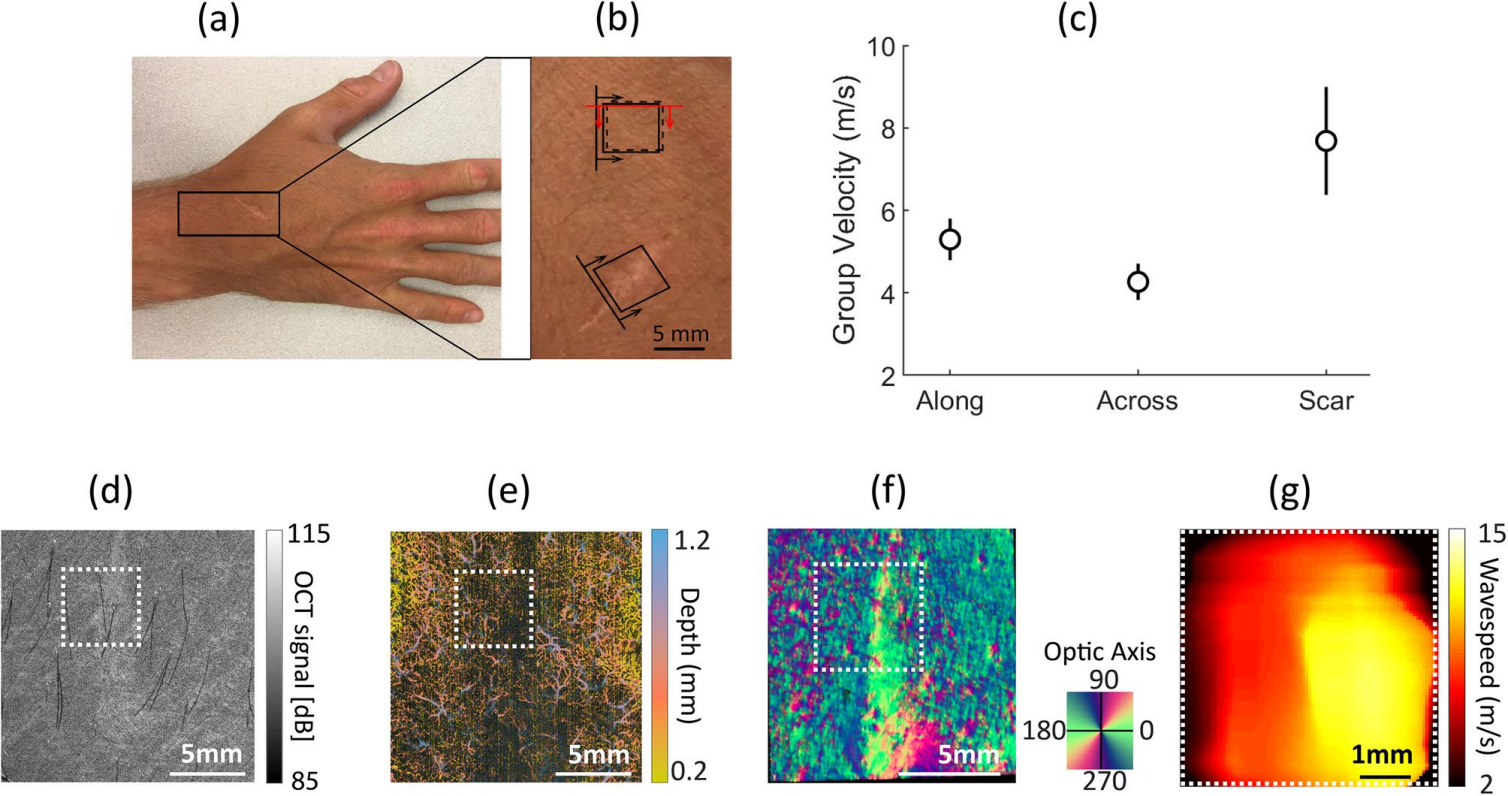
Characterization of scar tissue in vivo with four different OCT modalities. (**a**) Photograph of the scar area in a 28-year-old male volunteer. (**b**) Its enlarged area with the direction of mechanical wave propagation in the scar and adjacent skin site. (**c**) Group velocity of Rayleigh wave in scar and in normal skin tissue in two orthogonal directions. Structural OCT (**d**) and OCT angiography (**e**) images obtained with the system described in Ref.^72^. (**f**) Optic axis orientation map obtained with the PS-OCT system described in Ref.^68^. (**g**) Image of Rayleigh wave group velocity measured in the direction perpendicular to scar within the area covered by a white dashed rectangle (shown in panels (**d**–**f**) for scaling). The AμT-OCE image was obtained with the system described in Ref.^19,64,65^.

A 28 year old volunteer with a mature skin scar on the back of the hand (Fig. 5a,b) was imaged using different OCT modalities in the area around the scar. The Rayleigh wave speed was measured in the scar and compared with the wave speed in two orthogonal directions in normal tissue around the scar. The x and y scanning directions each had 100 scan locations, spread across a 6 mm × 6 mm scanning area. The wave speed was calculated using a moving kernel with 20 pixels. Overall, there were 80 different shear wave speed values (across x) by 100 values (across y) used to determine the mean and variation in Fig. 5c. As seen, the Rayleigh wave speed is almost twice higher in scar tissue, providing evidence of the increased density of collagen fibers in scar and with corresponding higher elastic moduli. This result is supported by the structural OCT image (Fig. 5d) where the scar area looks much brighter. The OCTa image (Fig. 5e) shows reduced density of capillaries, typical for certain types of mature scar tissue^71^. It can also be seen from the PS-OCT map (Fig. 5f) that the optic axis orientation is very different in scar compared to that of surrounding tissue. Finally, a 2D image of mechanical wave speed measured in the longitudinal direction in scar obtained with AμT-OCE is presented in Fig. 5g. It confirms very different mechanical properties of scar tissue with increased fiber content, and correlates well with the results of OCTa to demonstrate that mechanical properties of healed tissue cannot match those of surrounding areas without proper vascularization and collagen remodeling.

## Discussion

There is no doubt that elasticity is intimately related to skin’s aesthetics and function. Mapping skin’s functional and mechanical properties is critical for reconstructive surgeries to restore both form and function while minimizing the impact of scar formation.

Although there are several commercial mechanical devices to measure skin’s elastic properties, most use an inadequate, very simplified model of skin, cannot resolve spatial differences in stiffness parameters, and have limited use in assessing burns, traumas, and grafts. Traditional ultrasound elastography is also not appropriate because it requires mechanical contact to the measurement site and faces difficulties in moduli inversion from experimental data due to skin’s layered structure.

Current aesthetic and reconstructive procedures do not include mapping and monitoring of skin elasticity because there are no non-invasive devices providing quantitative maps of elastic properties. Thus, there are no quantitative methods to longitudinally monitor the reconstruction process and potentially generate feedback to help guide clinicians toward optimal outcomes and inform the timing for future reconstructive procedures. Serial evaluations of pathologic scars could also be used to document scar evolution over time and objectively measure improvements from non-surgical scar interventions such as CO2 laser remodeling.

In this paper, we proposed AμT-OCE (previously calibrated and tested for cornea) to map skin’s elastic moduli and anisotropy. The proposed method is non-contact and non-invasive. In addition, OCT is already FDA approved for clinical use including surgical applications. Thus, there is a clear, straightforward path to clinical translation of AμT-OCE if it can be shown to be accurate and efficient.

AμT-OCE has other advantages beyond being non-contact. First, it can be very fast. Modern OCT systems can scan a few million A-scans per second, which means OCE can be performed over a soft tissue area of ~ 1 cm^2^ in less than a second. The method, therefore, can be used for on-line monitoring and longitudinal diagnostics. Second, moduli inversion can be highly simplified in OCE compared to traditional elastography because AμT-OCE utilizes surface propagating waves. As demonstrated in Results and Supplementary Notes 5, 6, subcutaneous tissue may not greatly affect wave propagation in dermis due to both the gradual transition from dermis to hypodermis and the non-zero Young’s modulus of deeper tissue. Thus, a bulk NITI material is appropriate to reconstruct mechanical properties of the dermis from acoustic data. Third, all four OCT modalities (structural OCT, OCT angiography, PS-OCT and OCE) deliver important information on skin shape, structure, properties, and function. For example, skin thickness and its layered structure imaged with OCT can be used to refine the mechanical model for OCE. Vascularization maps from OCTa can be combined with skin’s elasticity for better characterization of scar and skin during reconstructive surgeries and aesthetic procedures. PS-OCT can help define skin’s anisotropy and differentiate lesions. It also delivers depth-resolved anisotropy, which is not currently demonstrated with OCE. Although we used different OCT setups to image skin with different OCT modalities, all four can be combined into a single device.

In our study, we used a NITI model to characterize the mechanical properties of skin. Although skin consists of 3 sequential layers, the epidermis functions mainly as a protective outer layer and does not greatly influence skin elasticity^54^. On the other hand, hypodermis mostly connects outer skin layers with internal structures and provides thermal insulation. Its elasticity is much smaller than that in dermis and, thus, dermis mainly defines skin mechanical properties. As discussed above, we showed that the Rayleigh wave speed used to reconstruct the Young’s modulus is mainly defined by the elastic properties of dermis. The thin epidermis is much smaller than the wavelength of propagating waves and does not significantly affect Rayleigh wave propagation. The results of fits to theoretical functions (see left column in Fig. 4) confirm that the NITI model well describes the anisotropy in dermis, even though the Rayleigh wave speed for in-plane anisotropy has a complicated shape. Finally, PS-OCT provided independent confirmation that the NITI model is appropriate for skin since the primary fiber orientation in dermis as measured by PS-OCT is very close to that obtained with AμT-OCE (see Fig. 4).

Although the results presented here on skin’s elasticity and its anisotropy in the human forearm are promising, future studies are clearly needed to validate its diagnostic value. Our studies here were limited to imaging forearm sites and acquiring pilot measurements in scar. We have not performed detailed studies of skin at other anatomical sites where its mechanical properties may be substantially different. For example, at sites in the face, and other areas where large blood vessels, bones or cartilages are located close to the skin surface, additional model refinement may be required. In addition, as PS-OCT images show (see Figs. 3, 4) in five volunteers, skin sites in the forearm have a primary fiber orientation (which can be different from subject to subject); however, it is not clear that skin at an arbitrary body site can be considered a NITI material. Thus, independent co-measurement of tissue anisotropy with PS-OCT, for example, or with any other method is desirable for all AμT-OCE studies. As shown in Fig. 5, the combination of different OCT modalities would be ideal because they can deliver very diverse information on skin’s structure (vascularization, skin layers’ thickness, in-depth distribution of optical anisotropy). All these parameters may help refine skin’s local biomechanical model and make reconstructing mechanical moduli more accurate and reliable.

In our NITI model (see Supplementary Notes 1–3), we ignored viscosity, which might influence reconstruction accuracy and require additional corrections. We observed that mechanical waves can propagate for more than 10 wavelengths over the skin surface for the anatomical sites studied and, therefore, viscosity is not very strong and does not significantly affect the propagation speed. If, indeed, guided waves are not excited in skin because of the smooth transition in elastic properties at the dermis/hypodermis interface, and the main mode is a Rayleigh wave, then skin’s viscosity can be determined from wave attenuation^73^. This will be a subject of future work.

Although the subjects in this study were age-matched and healthy, multiple factors can contribute to significant interpatient variability in the direction of the fiber orientation in dermis and its elastic moduli, even for the same anatomical location^74,75^. For example, for one human subject (Fig. 4a–c) results demonstrate higher elastic moduli and shear anisotropy compared to others, whereas in another subject (Fig. 4g–i), the primary fiber orientation in the dermis was different from what is typically assumed. These results highlight the importance of personalized elasticity measurements prior to interventions to improve surgical outcomes.

In future human subject studies, baseline elastic properties of skin will be obtained at different anatomical sites in volunteers of different age, gender, race, and body mass index. This information will be vital to define the normal range of variations in skin elastic properties and their influence on its aesthetics and function. Additionally, skin graft procedures may be monitored at all steps starting from initial mapping of skin’s elasticity in donor and recipient sites preoperatively, perioperatively, and postoperatively through the sequential wound healing phases of inflammation, proliferation, and remodeling. Finally, the existing AμT-OCE system must be optimized for clinical measurements. One such approach would be to fix the OCT head and AμT source on a 6-coordinate robotic arm with autofocus alignment. This would enable easy alignment of the imaging area to an arbitrary anatomical site while keeping the patient in a natural position. The imaging head could be rotated to carefully assess skin anisotropy at every measurement site. Both AμT-OCE and PS-OCT modalities can feasibly be a part of a single device.

We anticipate that a quantitative elasticity mapping tool appropriate for the clinic will dramatically improve skin reconstructive procedures by minimizing scars and optimizing outcomes. Similarly, we hope to show that longitudinal volumetric mapping of skin elasticity can reduce graft failure and secondary contracture at the recipient site and limit the need for revisionary surgery. We also hope that AμT-OCE can be utilized in future clinical studies to quantify the biomechanical function and aesthetic parameters of existing surgical procedures and aid in the development of new protocols for novel skin surgeries.

Mapping skin elasticity with sub-mm resolution is not limited to skin grafting only. It may significantly impact cosmetics, dermatology, transplantology and plastic surgery, dramatically improving current monitoring of wound healing and tissue recovery, reducing surgical failure rates, providing immediate quantitative feedback on all procedures, and opening many new opportunities for reconstructive medicine.

As a limitation of the method, our current 1 MHz AμT excitation cannot provide resolution better than 0.35 mm. Higher resolution may be required for some applications, such as split thickness graft surgery. Note, however, that resolution can be improved with a higher frequency AμT push.

## Methods

### Analytic model

Based on the presence of Langer’s lines defining the primary orientation of collagen fibers in skin, a NITI model was proposed to describe its elastic properties. As demonstrated in Results and in Supplementary Notes 4, 5, Rayleigh wave propagation in skin does not need to explicitly account for its layered structure, i.e. Rayleigh waves on the skin surface can be described as surface waves on a bulk NITI material (see Supplementary Notes 3). Propagation of bulk and Rayleigh mechanical waves are considered in Supplementary Notes 2 and 3, respectively.

### Numerical simulation

As noted above, the bulk NITI model was used to describe Rayleigh wave propagation over the skin surface. To study how a thin layer (epidermis) on the top of the NITI medium affects wave propagation, as well as how subcutaneous tissue influences guided wave behavior in dermis, numerical simulations were performed. We developed a finite element numerical model for both cases using OnScale (OnScale, Redwood City, CA). A full description of the simulation results is provided in Supplementary Notes 4 and 5.

The computational domain was discretized using linear finite elements on a regular rectangular grid with at least 40 elements per elastic wavelength. Simulations were solved using explicit time stepping, and the vertical velocity component was extracted for analysis, similar to OCE experiments where only this component is available.

OnScale scripts for the simulation of Rayleigh wave propagation in 2-layer (epidermis/dermis) and 3-layer (epidermis/dermis/subcutaneous tissue), as well as the corresponding Matlab scripts to compute and plot wavefields and 2D Fourier spectra of Rayleigh waves are provided in Supplementary Software Library.

Matlab scripts to calculate wave velocities of bulk and Rayleigh waves in the YZ plane of a fast-axis NITI material for different parameters $\mu$, $G,$ and $\delta$ are provided in Supplementary Software Library.

### AμT-OCE imaging system and measurement

To generate elastic waves without any contact to the skin surface, we applied an excitation push with acoustic micro-tapping (AμT), a technique using a cylindrically focused, air-coupled ultrasound transducer to induce a localized radiation force at the tissue surface^18–21,64,65^. The AμT transducer effectively applied a line load to the surface over a wide region relative to the propagation distance of interest, resulting in approximately planar elastic waves (normal to the OCT imaging plane).

**Figure 6:**
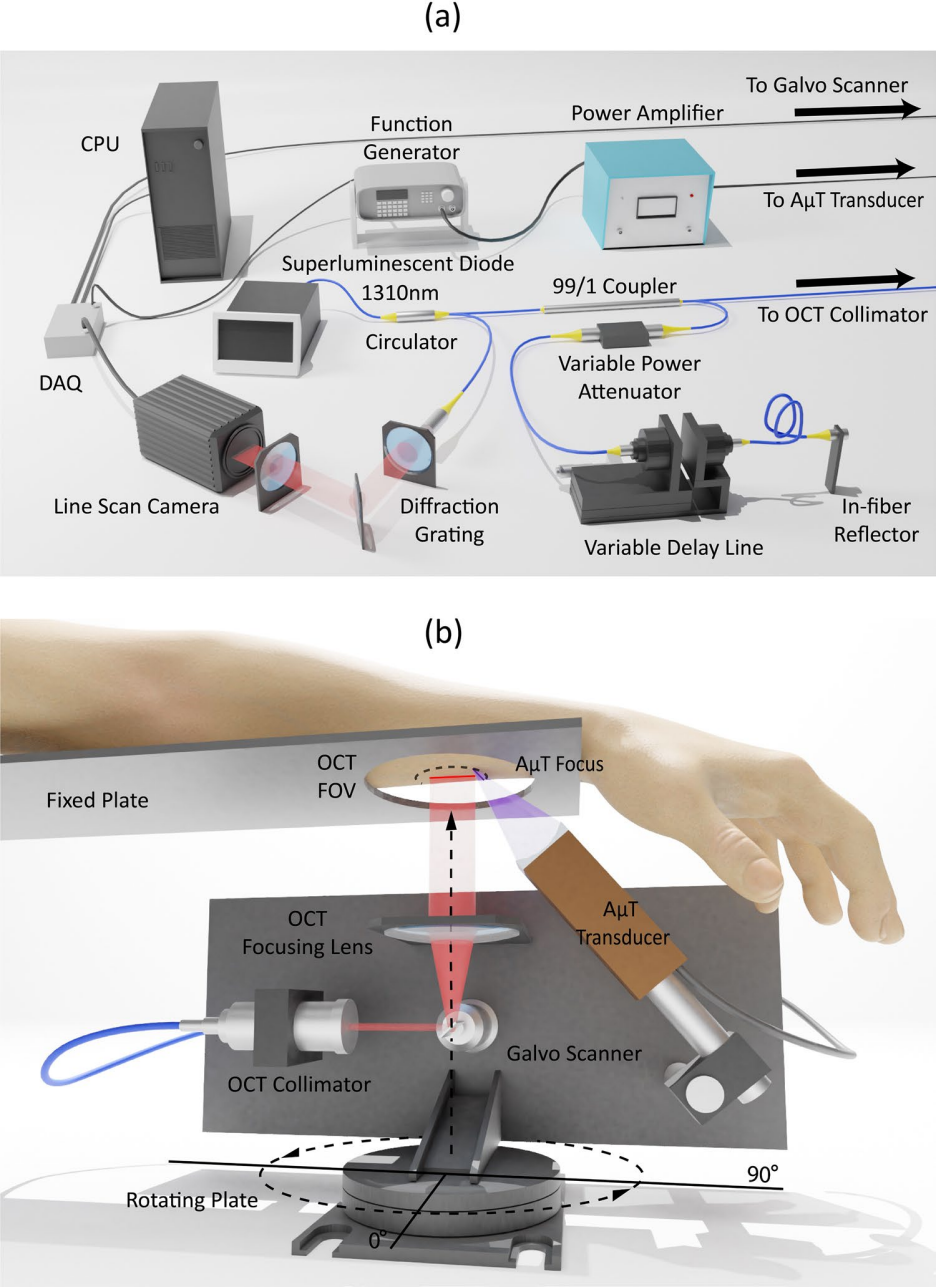
AμT-OCE measurement system: (**a**) schematic of a Fourier-domain OCT system utilizing polarization-maintaining fibers; (**b**) schematic of in vivo AμT-OCE measurement of mechanical wave propagation over the surface of skin for healthy volunteers. Rayleigh waves were excited with a cylindrically focused air-coupled transducer^18–20^. In-plane rotation of the measurement arm was used to track wave propagation in different directions over the skin surface.

Mechanical waves propagating over the skin surface were detected using a phase-sensitive frequency-domain OCT (PhS-OCT) system (see Fig. 6a), which has been described in previous studies^20,64,65^. The sampling rate of the 1024-pixel line-scan InGaAs array was set to 46.5 kHz, determining the A-line rate of the system (temporal resolution). The optical resolution was approximately 15 µm axially and 24 µm laterally. An external TTL trigger synchronized the PhS-OCT system with wave excitation for each M-scan. All data were collected in M-B format in which 512 A-scans are repeated in the same location (M-scan) at 256 different horizontal locations (B-scan) across the imaging plane (dx = 54.7 $\upmu$m), forming a complete M-B scan (1024 depth × 256 lateral locations × 512 temporal frames) with an effective imaging range of 1.5 mm × 10 mm (axial × lateral). One full M-B scan took 3.66 s.

To measure the angle-dependent surface wave speed, the imaging arm (consisting of a galvo mirror, focusing lens, and AμT transducer) was mounted on a rotational stage with the axis of rotation located in the center of the OCT field of view (FOV) (Fig. 6b). The rotational stage was equipped with an adjustable screw that enabled fine-angle rotations over a range of 180°. The entire imaging arm was rotated 15° at a time to measure wave propagation along different directions. A 20 mm wide rigid circular ring was mounted near the co-aligned OCT and AμT focus to ensure easy forearm alignment and limit bulk motion.

### Reconstruction of Young’s modulus and elastic anisotropy in skin

AμT-OCE recorded Rayleigh wavefields (see Fig. 2c–i) propagating over the skin surface at different orientations relative to the longitudinal forearm direction. Linear fits were applied to all wavefields to obtain the group velocity of propagating waves. Wave velocities measured at different propagation directions created the anisotropy map of surface wave speed.

Ideally, to reconstruct elastic moduli, an analytic solution for the Rayleigh wave equation should be used. Unfortunately, obtaining an analytic form of the Rayleigh wave equation in the NITI medium is possible only along the primary symmetry axes^76^ (see Supplementary Note 3), which is not sufficient to reconstruct all 3 constants.

Instead, experimentally obtained wave speed anisotropy maps were fit with numerically obtained functions of Rayleigh wave anisotropy in the NITI model. Fitting was performed with four parameters: $G$, $\mu$ and $\delta$ and fiber orientation $\alpha$. The last had an original guess corresponding to the results obtained with the PS-OCT for the fiber orientation in dermis. The accuracy of reconstruction and its sensitivity to experimental data variation was determined by a ‘Leave-one-out cross-validation’ method^66^. An example of fitting results is presented in Fig. 2b; summary of data fitting for all human subjects is shown in Fig. 4.

### PS-OCT imaging system and measurement

The PS-OCT system^69,70^ used a 100-kHz MEMS-VCSEL swept laser source (SL1310V1-20048, Thorlabs), providing an output power of 25 mW with a central wavelength of 1310 nm and a spectral tuning range of 100 nm. The source output was sent to a polarization controller and linearly polarized through a polarization beam splitter and then split into reference and sample arms through an additional beam splitter at a split-ratio of 50:50. The sample arm was equipped with a quarter wave plate (QWP) aligned at 45° with respect to the input polarization state, resulting in a circularly polarized light incident at the sample surface. Light coming back from both reference and sample arms were recombined so that interfering light was split into horizontal and vertical polarization components. Balanced detection was used for both channels to collect the interference signals, from which PS-OCT images are reconstructed. In this system, PS-OCT was performed with the objective lens (LSM03, Thorlabs Inc., U.S.), providing a lateral resolution of 30 μm. The axial resolution was approximately 7.5 μm, in air.

The FOV was 11 mm × 11 mm centered over the OCE region marked with ink. To improve SNR, four repeat B-scans were performed at each location and interference signals averaged prior to processing. Entire volumetric scanning, consisting of 500 A-lines in the y-axis direction and 2000 A-lines in the x-axis direction, took 13.2 s.

### Reconstruction of depth-resolved orientation of optic polarization axis in skin

A polarization state tracing (PST) method was used to derive the depth-resolved phase retardation and axis orientation from PS-OCT measurements^70^. In this approach, output polarization states were represented by the Stokes parameters^68^. Before depth-resolved phase retardation and axis orientation were calculated, a color filter was applied to remove amorphous tissue at the surface that does not alter input polarization^68^. A sliding window containing 3 adjacent output polarization states along depth was used to do local plane fitting progressively using singular value decomposition. Then, the normal vector of each local fitting plane is obtained and utilized to determine the final spatially resolved orientation of the optic polarization axis^70^.

### OCT/OCT angiography imaging system and measurement

The swept-source OCT (SS-OCT) system employed in this study has been previously reported in detail^77^. Briefly, a 200-kHz vertical-cavity surface-emitting (VCSEL) swept laser source (SL1310V1-10048, Thorlabs Inc., Newton, NJ, USA) with a central wavelength of 1305 nm (spectral tuning range of 100 nm) was utilized, providing an axial resolution of ~ 8 μm in tissue (~ 11 μm in air). The imaging arm was attached to a hand-held probe via shielded optical cables and an 18 mm effective focal length lens (LSM02, Thorlabs Inc.) was used to provide a lateral resolution of ~ 10 μm. A glass coverslip was attached to ensure the OCT focal plane was aligned with the epidermal-dermal junction and provide stability during imaging. A small amount of glycerol was applied to the skin to remove air gaps between the glass and skin.

3D volumetric scans were acquired with a FOV of 10 mm × 10 mm. The beam spot was scanned using a paired X–Y galvo scanner (6210H, Cambridge Technology, Bedford, MA, USA), forming raster sampling patterns comprising fast (x-axis) and slow (y-axis) scans. At each y-location, 800 A-scans were acquired to create a single B-frame. Eight B-frames were repeated before moving to the next y-location to improve SNR. Following this protocol, a single 3D volumetric scan (C-scan) was generated (a detailed analysis of a typical OMAG scan sequence was previously reported by Deegan et al*.*^72^).

Repeated frames were used to generate the optical microangiography (OMAG) image based on eigen decomposition (ED) analysis^78^. This technique uses repeated B-frames to provide a 3D volume image contrasted by particles in motion. The scan protocol was designed to contrast capillary vessels based on red blood cell scattering, providing 3D contrast of vascular structure. The method resulted in a co-registered image of both static (tissue) and dynamic (RBC) components providing information on both local tissue and vascular structure. For visualization, 3D data were compressed to maximum intensity projected en-face vascular images, with the ED-signal above a 5 dB threshold displayed and mapped to a color based on depth^72^. 3D data were cropped approximately 450 μm below the tissue surface to limit the noise-contribution from light attenuation.

### Human subjects

The study was performed in accordance with University of Washington policies and regulation applied to the studies involving human subjects and approved by the University of Washington Institutional Review Board (IRB) (STUDY00012306). University of Washington IRB was established in accordance with the federal regulations for protecting the rights and welfare of human research subjects. For all volunteers who participated in this study, informed consent was obtained.

Five healthy volunteers between 28 and 32 years old (2 male, 3 female), nonsmokers without known skin conditions and diseases, were scanned in vivo with both AμT-OCE and PS-OCT systems in the forearm area. A small piece of black tape was attached to the subjects’ skin to align both AμT-OCE and PS-OCT measurements. A black felt-tip marker highlighted the ROI used in both OCE and PS-OCT.

Additional measurements with four different OCT modalities (structural OCT, OCTa, AμT-OCE and PS-OCT) were performed around scar tissue located on the back of the hand of a 28-year-old male volunteer. The mature scar tissue formed during the healing process of a deep incision wound. The wound was allowed to heal naturally (no sutures) resulting in a ~ 11 mm long by ~ 4 mm wide region of scar tissue. An imaging window was carefully cleared using a shaving razor 24 h prior to imaging.

AμT-OCE and PS-OCT measurements were performed in vivo without mechanical contact or any skin preparations to subjects. Structural OCT and OCTa utilized a glass coverslip to reduce motion artifact but can be performed without contact following small system modification.

## Supplementary Information

Supplementary Information 1.

Supplementary Information 2.

## Data Availability

The authors declare that all data from this study are available within the Article and its Supplementary Information. Raw data for the individual measurements are available on reasonable request.
