# Supplementary figures and images for: Probing elastic anisotropy of human skin in vivo with light using non-contact acoustic micro-tapping OCE and polarization sensitive OCT
Source: Sci Rep. 2022 Mar 10;12:3963. doi: 10.1038/s41598-022-07775-3 (PMC8913799; doi:10.1038/s41598-022-07775-3)

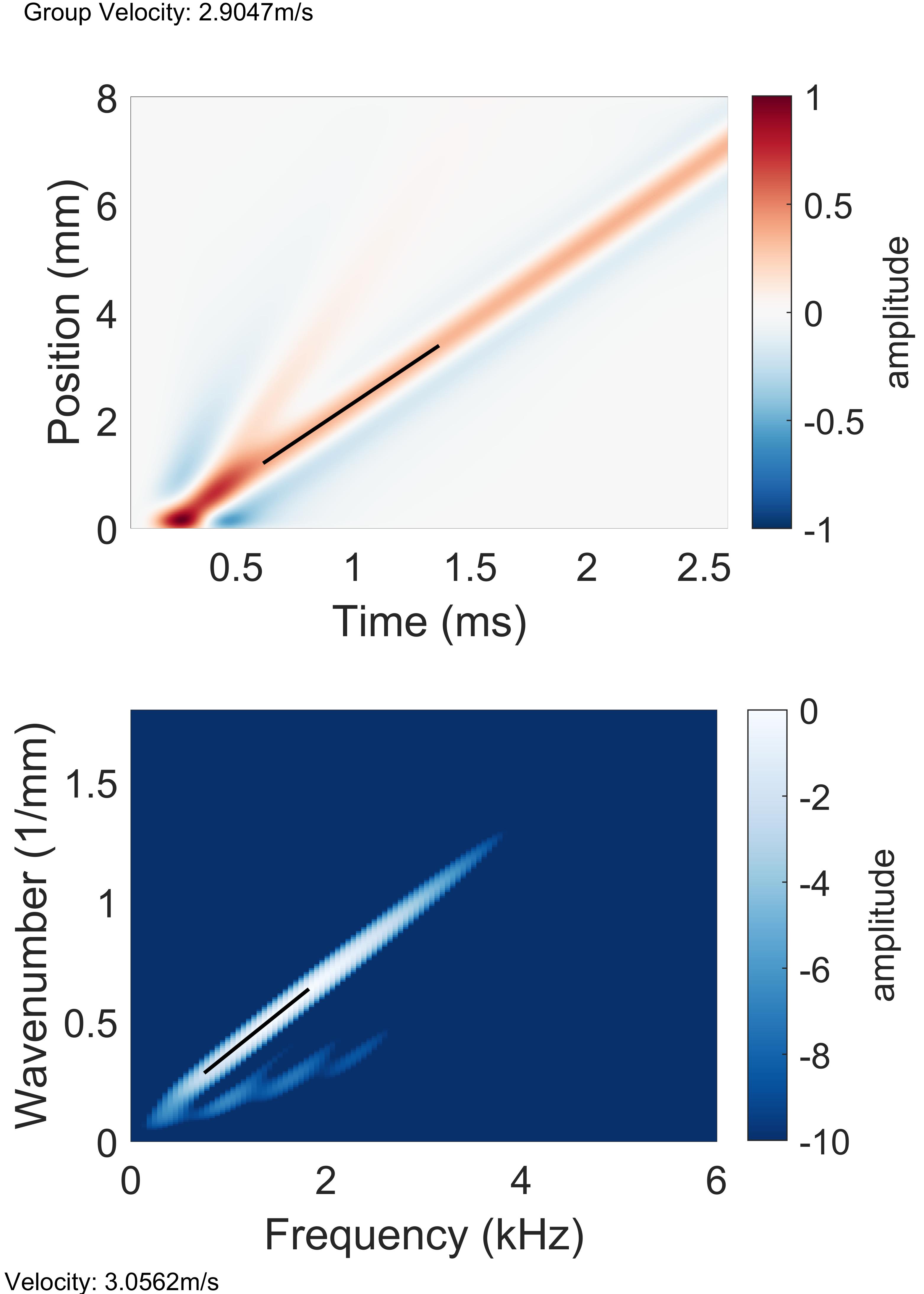

Supplement: Supplementary file 1 — Supplementary Information 1. [file 41598_2022_7775_MOESM1_ESM.zip › Supplementary Sortware Library/OnscaleDataPlotting/finalResults/1Layer/across.jpg]

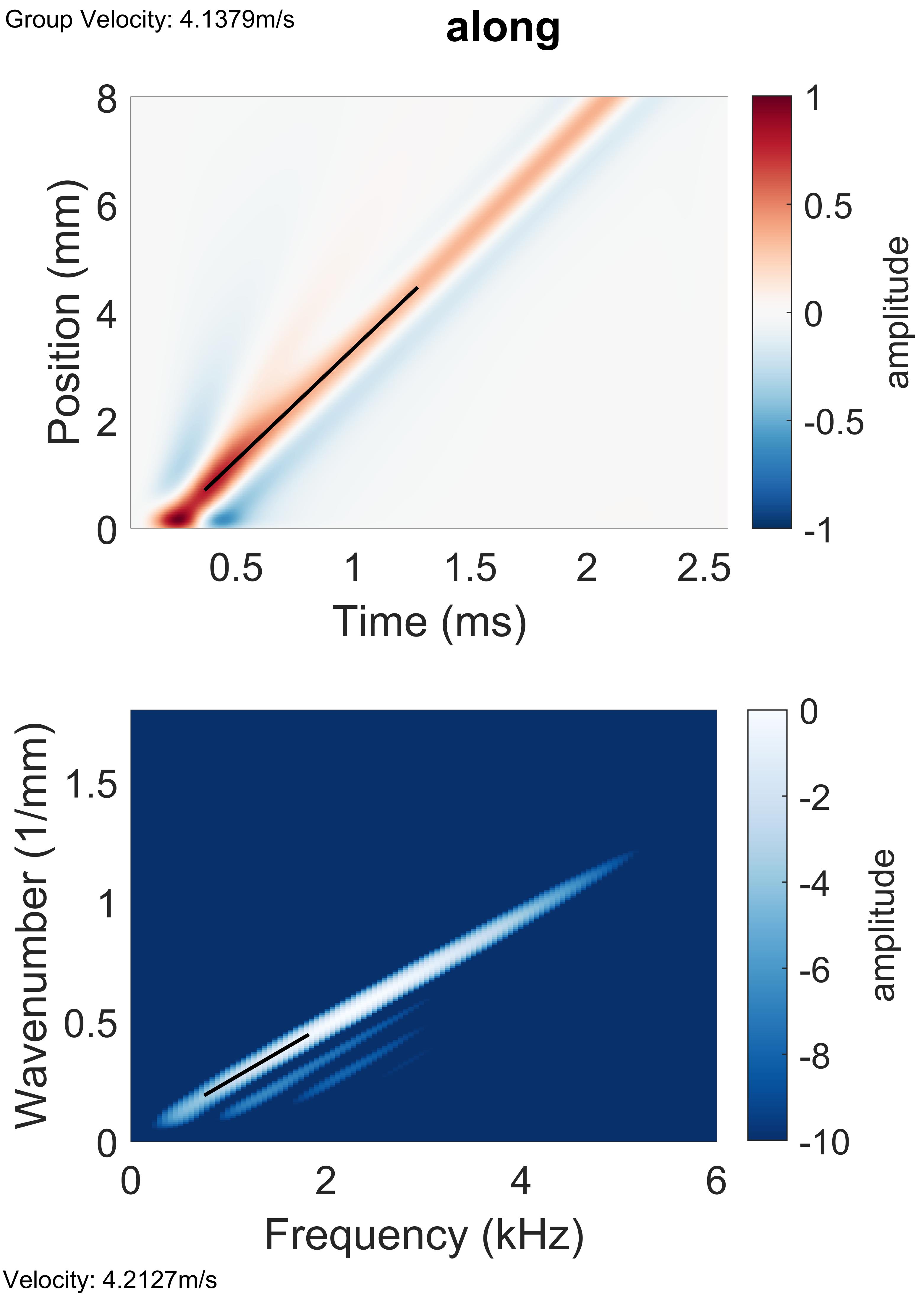

Supplement: Supplementary file 1 — Supplementary Information 1. [file 41598_2022_7775_MOESM1_ESM.zip › Supplementary Sortware Library/OnscaleDataPlotting/finalResults/1Layer/along_xt_fk.jpg]

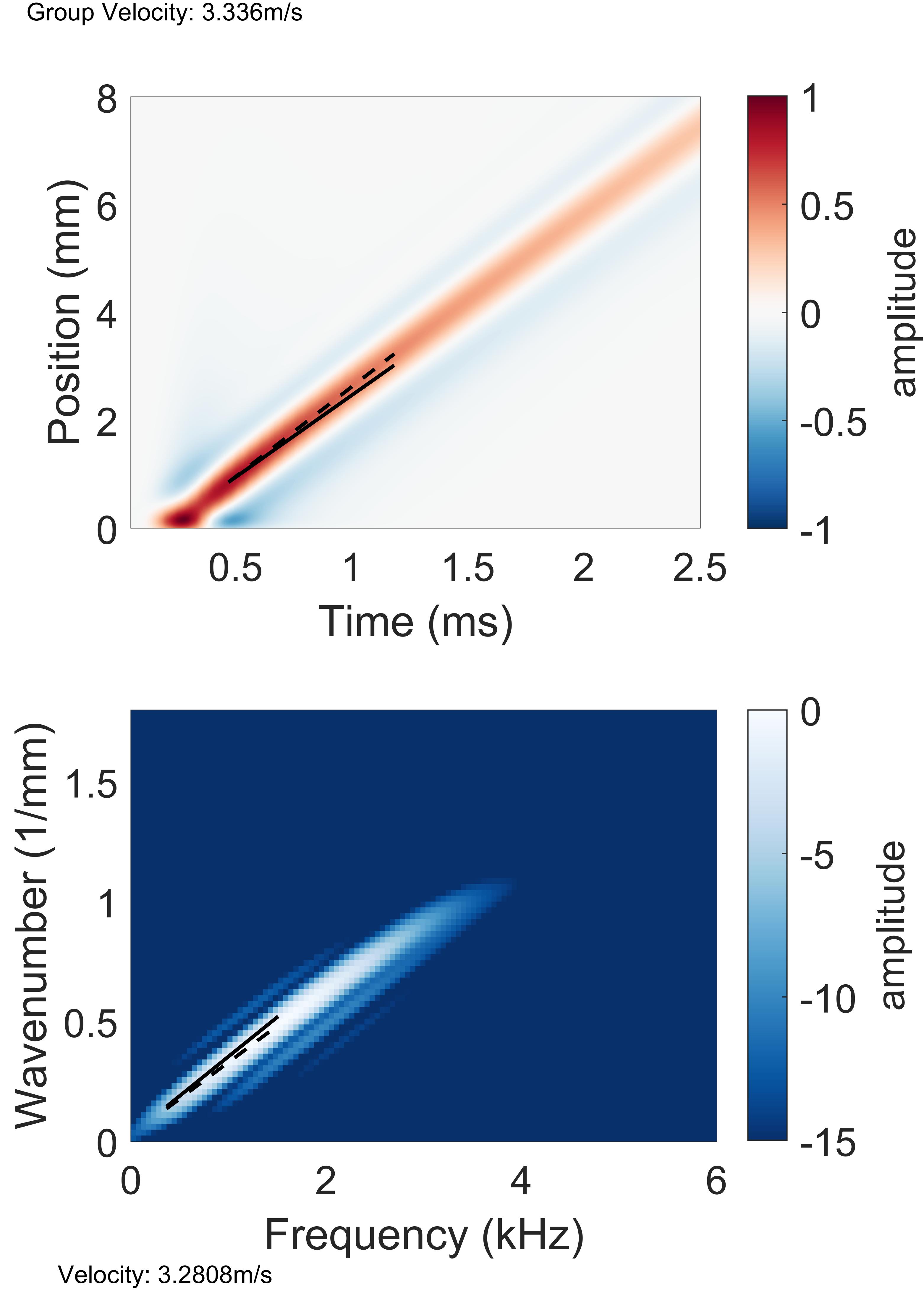

Supplement: Supplementary file 1 — Supplementary Information 1. [file 41598_2022_7775_MOESM1_ESM.zip › Supplementary Sortware Library/OnscaleDataPlotting/finalResults/2Layer/across.jpg]

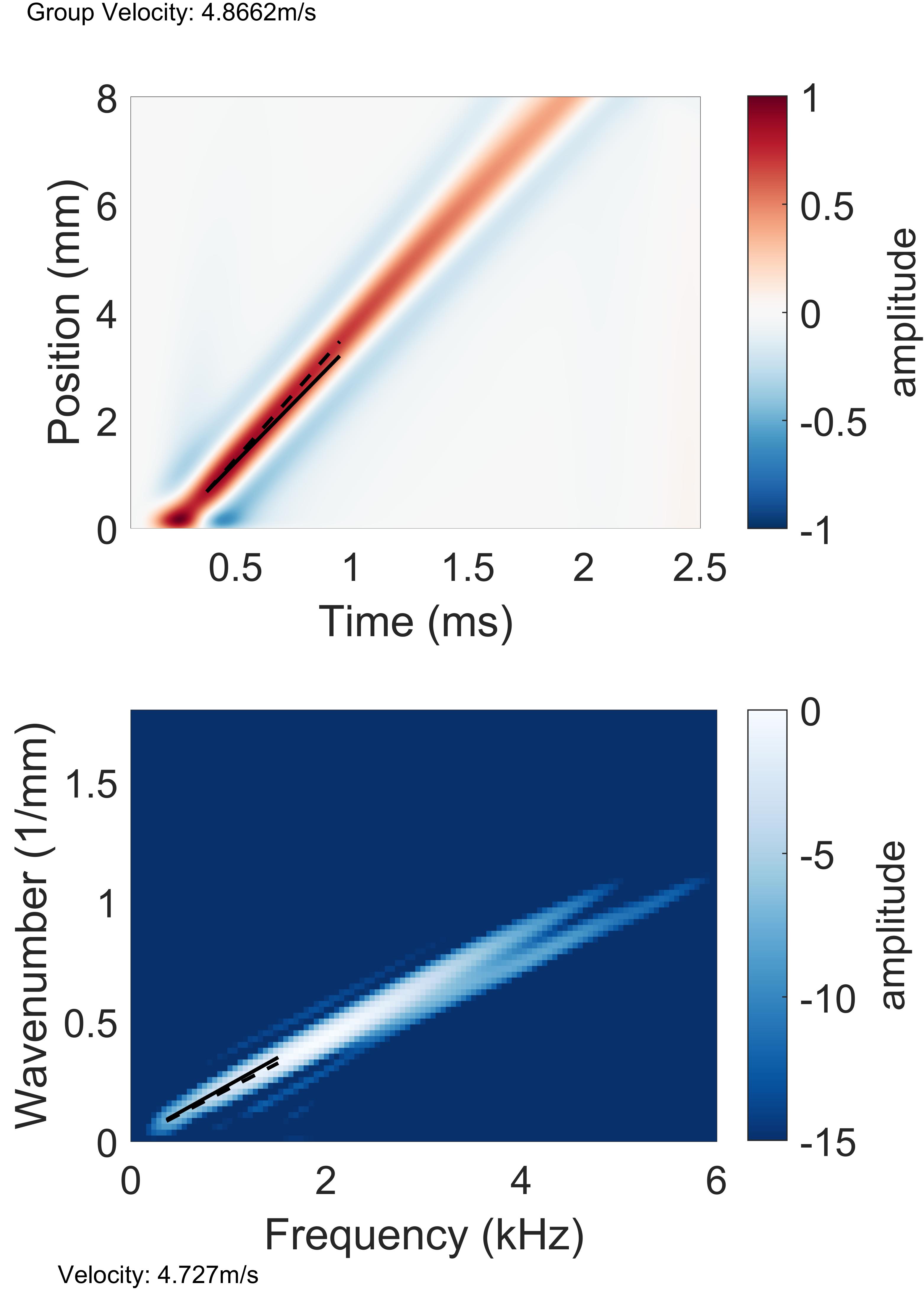

Supplement: Supplementary file 1 — Supplementary Information 1. [file 41598_2022_7775_MOESM1_ESM.zip › Supplementary Sortware Library/OnscaleDataPlotting/finalResults/2Layer/along.jpg]

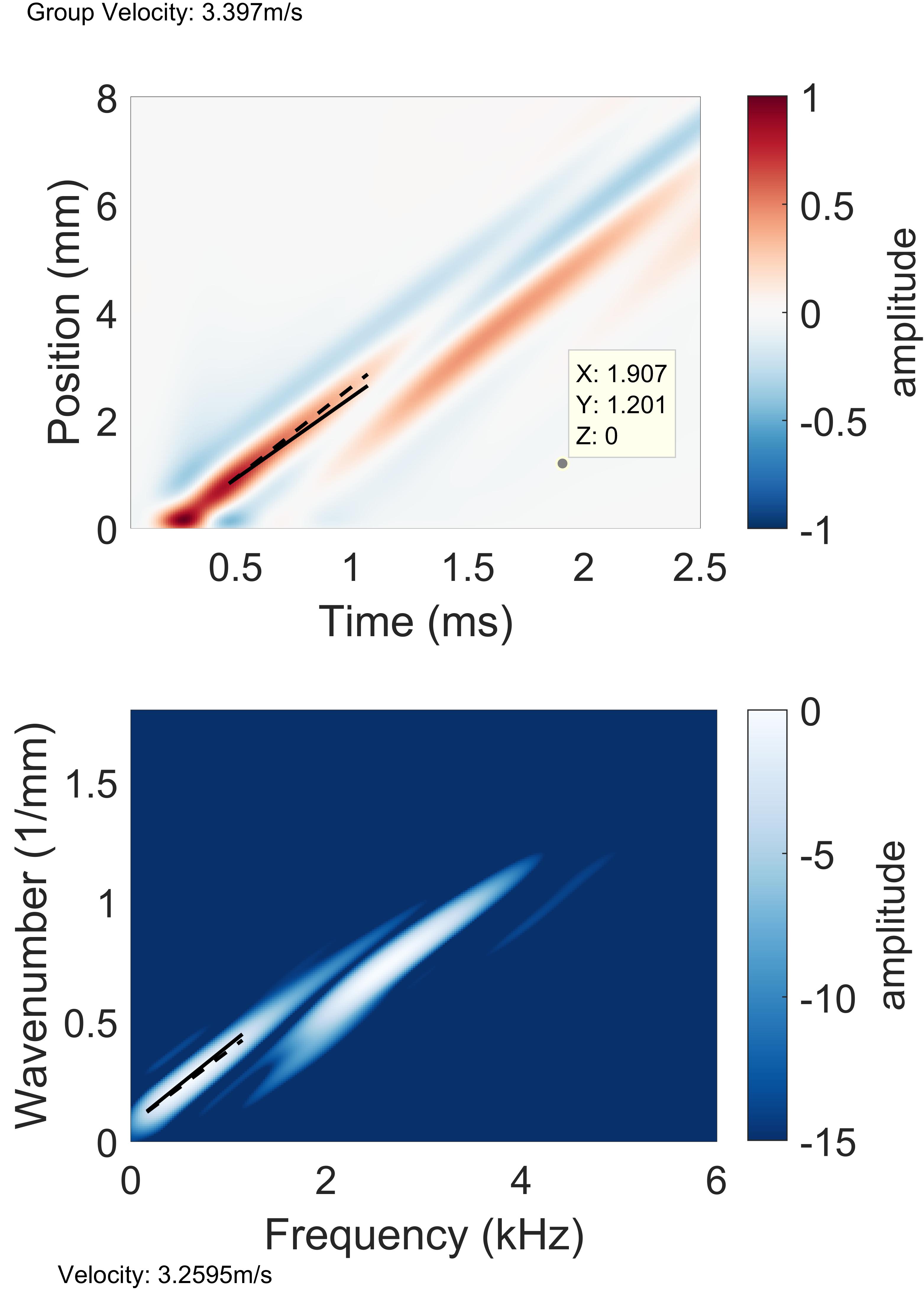

Supplement: Supplementary file 1 — Supplementary Information 1. [file 41598_2022_7775_MOESM1_ESM.zip › Supplementary Sortware Library/OnscaleDataPlotting/finalResults/3Layer/across.jpg]

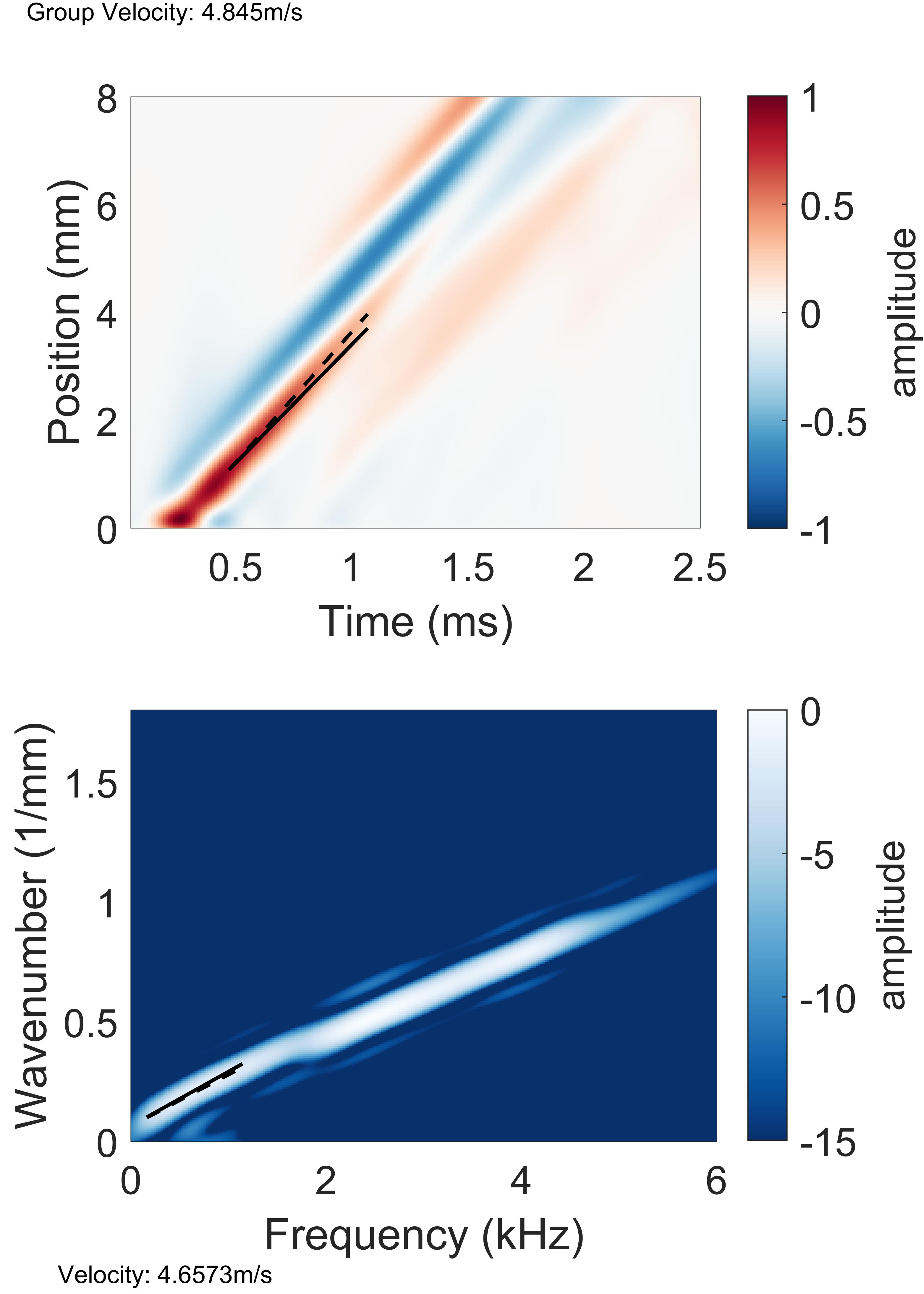

Supplement: Supplementary file 1 — Supplementary Information 1. [file 41598_2022_7775_MOESM1_ESM.zip › Supplementary Sortware Library/OnscaleDataPlotting/finalResults/3Layer/along5_right.jpg]
