# Supplementary material for: Probing elastic anisotropy of human skin in vivo with light using non-contact acoustic micro-tapping OCE and polarization sensitive OCT
Source: Sci Rep. 2022 Mar 10;12:3963. doi: 10.1038/s41598-022-07775-3 (PMC8913799; doi:10.1038/s41598-022-07775-3)
Supplement: Supplementary file 2 — Supplementary Information 2. [file 41598_2022_7775_MOESM2_ESM.pdf]

Supplementary Notes

Probing elastic anisotropy of human skin in vivo with light using non-contact acoustic micro-tapping OCE and polarization-sensitive OCT

*Mitchell A. Kirby [1], Peijun Tang [1], Hong-Cin Liou [1], Maju Kuriakose [1], John J. Pitre Jr.[1], Tam Pham [2], Russell E. Ettinger [2], Ruikang K. Wang [1], Matthew O'Donnell [1], and Ivan Pelivanov [1]**

[1.] University of Washington, Department of Bioengineering, Seattle, Washington, United States

[2.] University of Washington, Harborview Medical Center, Seattle, Washington, United States

Supplementary Note 1. Elastic constants of a linear elastic, nearly incompressible, transversely isotropic (NITI) material

The elastic modulus matrix of a linear-elastic, transversely isotropic (TI) material can be represented in a form [1, 2]:

$$C = \begin{bmatrix} C_{11} & C_{12} & C_{13} & & & \\ C_{12} & C_{11} & C_{13} & & & \\ C_{13} & C_{13} & C_{33} & & & \\ & & & C_{44} & & \\ & & & & C_{44} & \\ & & & & & C_{66} \end{bmatrix}, \tag{S1}$$

where $C_{12} = C_{11} - 2C_{66}$ due to symmetry conditions [1, 2]. Considering that two of three principal planes in a TI material are isotropic, we will use a modified notation of moduli based on the notation common of isotropic materials [3]:

$$C = \begin{bmatrix} \lambda + 2\mu & \lambda & \lambda + Q_1 & & & \\ \lambda & \lambda + 2\mu & \lambda + Q_1 & & & \\ \lambda + Q_1 & \lambda + Q_1 & \lambda + 2\mu + Q_2 & & & \\ & & & G & & \\ & & & & G & \\ & & & & & \mu \end{bmatrix}. \tag{S2}$$

An additional modulus G shows that shear deformation can be different if shear stress is applied along the symmetry axis $\mathbf{z}$ compared to that applied across it. Parameters Q_1 and Q_2 indicate that differences between C_{33} and C_{11}, and between C_{12} and C_{13} may be nonzero [3].

Using the elasticity matrix (S.2), the stress-strain relation in a TI material for small deformations is described by Hook's law and takes the form (in Voigt notation) [3]:

$$\begin{bmatrix}\sigma_{11}\\ \sigma_{22}\\ \sigma_{33}\\ \sigma_{23}\\ \sigma_{31}\\ \sigma_{12}\end{bmatrix}=\begin{bmatrix}\lambda+2\mu & \lambda & \lambda+Q_1 & & & \\ \lambda & \lambda+2\mu & \lambda+Q_1 & & & \\ \lambda+Q_1 & \lambda+Q_1 & \lambda+2\mu+Q_2 & & & \\ & & & G & & \\ & & & & G & \\ & & & & & \mu\end{bmatrix}\begin{bmatrix}\varepsilon_{11}\\ \varepsilon_{22}\\ \varepsilon_{33}\\ 2\varepsilon_{23}\\ 2\varepsilon_{31}\\ 2\varepsilon_{12}\end{bmatrix}, \tag{S3}$$

where σ_{ij} and ε_{kl} are the components of the stress and strain tensors, respectively.

For any arbitrary load applied to a TI medium, the strain field is computed by inverting the stress-strain relation, where the inverse of the elastic modulus matrix is the compliance matrix $\alpha = C^{-1}$:

$$\begin{bmatrix}\varepsilon_{11}\\ \varepsilon_{22}\\ \varepsilon_{33}\\ 2\varepsilon_{23}\\ 2\varepsilon_{31}\\ 2\varepsilon_{12}\end{bmatrix}=\frac{1}{\Delta}\begin{bmatrix}\alpha_{11} & -\alpha_{12} & -\alpha_{13} & & & \\ -\alpha_{12} & \alpha_{11} & -\alpha_{13} & & & \\ -\alpha_{13} & -\alpha_{13} & \alpha_{33} & & & \\ & & & \Delta\cdot\alpha_{44} & & \\ & & & & \Delta\cdot\alpha_{44} & \\ & & & & & \Delta\cdot\alpha_{66}\end{bmatrix}\begin{bmatrix}\sigma_{11}\\ \sigma_{22}\\ \sigma_{33}\\ \sigma_{23}\\ \sigma_{31}\\ \sigma_{12}\end{bmatrix}, \tag{S4}$$

where the components of the compliance matrix α_{ij} can be expressed in the form:

$$\alpha=\begin{bmatrix}1/E_T & \frac{-\nu_{TT}}{E_T} & \frac{-\nu_{LT}}{E_L} & & & \\ \frac{-\nu_{TT}}{E_T} & 1/E_T & \frac{-\nu_{LT}}{E_L} & & & \\ \frac{-\nu_{LT}}{E_L} & \frac{-\nu_{LT}}{E_L} & 1/E_L & & & \\ & & & 1/G & & \\ & & & & 1/G & \\ & & & & & 1/\mu\end{bmatrix}, \tag{S5}$$

where E_T and E_L are the transversal and longitudinal Young's moduli, respectively, and ν_{TT} and ν_{LT} are Poisson's ratios, respectively. These parameters can be expressed through the components of the compliance matrix as [3]:

$$
\begin{aligned}
E_T &= \frac{\Delta}{\alpha_{11}}, \\
E_L &= \frac{\Delta}{\alpha_{33}}, \\
\nu_{TT} &= \frac{\alpha_{12}}{\alpha_{11}}, \\
\nu_{TL} &= \frac{\alpha_{13}}{\alpha_{11}} \\
\nu_{LT} &= \frac{\alpha_{13}}{\alpha_{33}},
\end{aligned}
\tag{S6}
$$

where

$$
\begin{aligned}
\Delta &= 4\mu\left(\left(\lambda + 2\mu + Q_2\right)(\lambda + \mu) - \left(\lambda + Q_1\right)^2\right), \\
\alpha_{11} &= \left(\lambda + 2\mu + Q_2\right)(\lambda + 2\mu) - \left(\lambda + Q_1\right)^2, \\
\alpha_{12} &= \lambda\left(\lambda + 2\mu + Q_2\right) - \left(\lambda + Q_1\right)^2, \\
\alpha_{13} &= 2\mu\left(\lambda + Q_1\right), \\
\alpha_{33} &= 4\mu(\lambda + \mu).
\end{aligned}
\tag{S7}
$$

We also note also that $\frac{\nu_{LT}}{E_L} = \frac{\nu_{TL}}{E_T}$.

The incompressibility condition appropriate for materials such as soft tissue constrains the strain such that

$$
Tr(\boldsymbol{\varepsilon}) \equiv \theta = 0\,. \tag{S8}
$$

For an accurate description of an incompressible TI material, this condition must be applied directly to the stress strain relations in the limit that θ approaches zero and the longitudinal modulus λ approaches infinity, i.e.

$$
P = \lim_{\substack{\lambda \to \infty \\ \theta \to 0}} (\lambda\theta)\,, \tag{S9}
$$

where P is defined as the scalar pressure. As demonstrated in Ref [3], a strict application of the incompressibility condition produces stress-strain relations in which the longitudinal terms include the scalar pressure P and inversion of this equation to compute the Young's moduli and Poisson's ratios of an incompressible TI material is not strictly defined. However, following an approach presented in Ref [3] for an isotropic incompressible material, the Young's moduli and Poisson's ratios can be approximated using the formulas presented above and taking the limit as λ approaches infinity. Under these conditions, the Young's moduli and Poisson's ratios for a nearly-incompressible TI material, i.e. NITI material, can be approximated as:

$$
\begin{aligned}
E_T &= 3\mu + \mu\left[\frac{\delta}{4\mu+\delta}\right], \\
E_L &= 3\mu + \delta, \\
\nu_{TT} &= \frac{1}{2}\left[1 + \frac{\delta}{4\mu+\delta}\right] = 1 - \frac{1}{2}\frac{E_T}{E_L}, \\
\nu_{TL} &= \frac{1}{2}\left[1 - \frac{\delta}{4\mu+\delta}\right] = \frac{1}{2}\frac{E_T}{E_L}, \\
\nu_{LT} &= \frac{1}{2},
\end{aligned}
\tag{S10}
$$

where $\delta = Q_2 - 2Q_1$.

Note that in the limit of an isotropic material, where $Q_1 = Q_2 = 0$, $E_T = E_L = 3\mu$, and $\nu_{TT} = \nu_{TL} = \nu_{LT} = 1/2$.

We will further differentiate a fast-axis NITI material (i.e. the material with a unidirectional fiber orientation and, thereby, the symmetry axis collinear with fibers) considered in this paper for skin, from a slow-axis NITI material (i.e. the material where fibers arbitrary distributed in a plane and, thereby, the symmetry axis is perpendicular to the fiber direction) previously introduced for cornea [4].

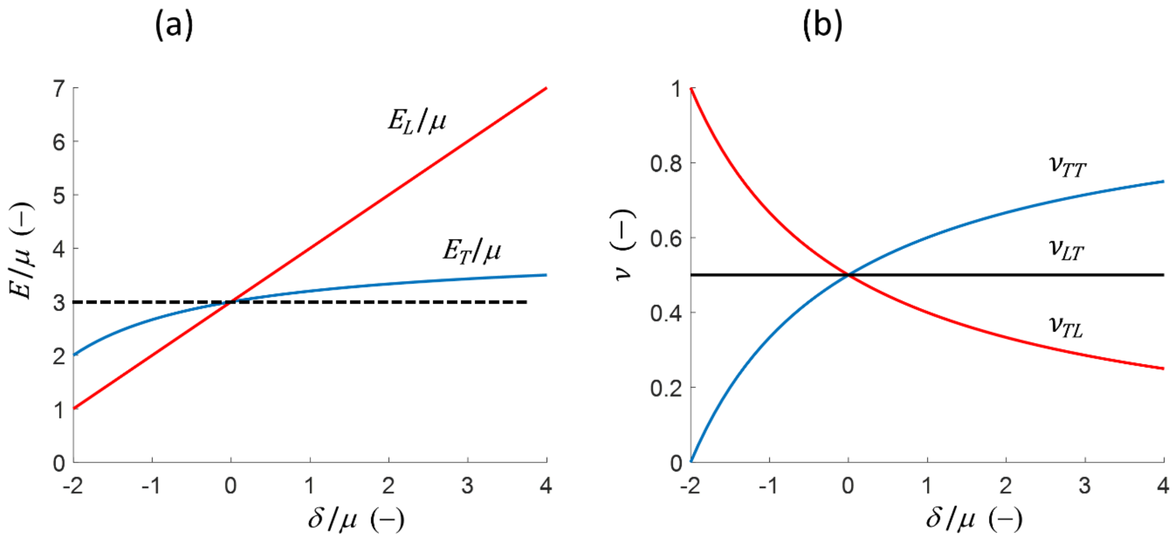

Supplementary Figure S1. Young's moduli **(a)** and Poisson's ratios **(b)** along and across NITI material symmetry axis **z** (fiber direction) scaled to the shear modulus μ (E_L/μ and E_T/μ respectively) as a function of the ratio δ/μ. Dashed line in panel **(a)** corresponds to the isotropic case of $E_T = E_L = 3\mu$.

Supplementary Figure 1 shows how Young's moduli and Poisson's ratios can change with δ. Clearly, E_T has a very narrow range, with a lower limit of 2μ and upper limit of 4μ as $\delta \to \infty$, and E_L is a linear function of δ.

A lower limit for the argument δ/μ is defined by the restriction for Poisson's ratio ν_{TT} to be positive.

The case when $\delta/\mu < 0$ corresponds to the slow-axis NITI medium, when $C_{33} < C_{11}$. This situation is realized in cornea, when lamellae are oriented randomly in-plane of the cornea, and, therefore, the symmetry Z-axis corresponds to the direction perpendicular to fibers.

The case when $\delta/\mu > 0$ corresponds to the fast-axis NITI medium, when $C_{33} > C_{11}$. This situation is typical for skin and muscle, where fibers can be considered locally unidirectional, and, therefore, the symmetry Z-axis corresponds to the direction along the fibers.

Supplementary Note 2. Bulk waves in a linear elastic, fast-axis NITI material

An isotropic solid supports two unique waves – one longitudinal with speed c_L and one shear with speed c_S. Biological tissue is nearly incompressible, meaning that the longitudinal wave speed (typically ≈ 1540 m/s) is much larger than the shear wave speed (typically ≈ 1-10 m/s).

In biological tissue such as the skin, the natural orientation of collagen fibers in the dermis are traditionally described using Langer's lines and correspond with estimates of mechanical anisotropy. Thus, in skin, a single shear modulus is not sufficient to describe elastic behavior. The simplest constitutive model that may account for skin's structure is a NITI model, recently introduced for describing elastic anisotropy in cornea [4]. In cornea, however, the orientation of fibers is in plane but quite random. This creates a symmetry axis perpendicular to the fiber structure. Thus, the symmetry axis for cornea is a slow axis. In contrast, the orientation of collagen fibers in skin is locally unidirectional, which creates a symmetry axis (which is the fast axis) in the plane of skin.

First, we consider propagation of bulk acoustic waves in a fast-axis NITI medium in the YZ plane (see Fig S2a below), parallel to the skin surface, but within the skin volume. Wave speeds can be found from the solution of the Christoffel equation [2]:

$$[\Gamma_{il} - \rho c^2 \delta_{il}]\mathbf{a} = 0 \tag{S11}$$

where $\Gamma_{il} = C_{ijkl} m_j m_k$ is the Christoffel tensor; $m_{j,k}$ $(j, k = 1, 2, 3)$ are the directional cosines of the propagation vector $\mathbf{r}$; δ_{il} is the Kronecker operator; $\mathbf{a}$ is the wave polarization unit vector, which is also the eigenvector of tensor Γ_{il}; and c is the velocity of the propagating mechanical wave.

In the YZ plane, the unit vector for directional cosines takes the form $m_{j,k} = [0, \sin\vartheta, \cos\vartheta]$. In this plane, the solution points to three bulk waves: a quasi-longitudinal, a quasi-shear (c_{qS}), and a shear wave, that propagate with speeds c_{qL}, c_{qS} and c_S, respectively [5]:

$$c_{qL,S} = \sqrt{\frac{C_{11}\sin^2\vartheta + C_{33}\cos^2\vartheta + C_{44} \pm \sqrt{M(\vartheta)}}{2\rho}}\ , \tag{S12}$$

where

$$M(\vartheta) = [(C_{11} - C_{44})\sin^2\vartheta + (C_{44} - C_{33})\cos^2\vartheta]^2 + (C_{13} + C_{44})^2 \sin^2 2\vartheta\ , \tag{S13}$$

$$c_S = \sqrt{\frac{G\cos^2\vartheta + \mu\sin^2\vartheta}{\rho}}\ . \tag{S14}$$

Velocities c_{qL} or c_{qS} can be calculated using the positive or negative discriminants, respectively. Because $\lambda \gg \mu, G, \delta$ in nearly incompressible media, variations of quasi-longitudinal wave speed

c_{qL} are negligible over all angles and can be approximately set as $c_L = \sqrt{\lambda/\rho}$, and the speed c_{qS} of quasi-shear wave can be expressed as:

$$c_{qS} = \sqrt{\frac{G + (\mu + \delta/4 - G)\sin^2 2\vartheta}{\rho}} = \sqrt{\frac{G + \left(\frac{E_L}{E_T}\mu - G\right)\sin^2 2\vartheta}{\rho}}. \quad \text{(S15)}$$

The shear wave is always polarized along the X-axis. It can also be shown, that in the near-incompressible limit, the polarization of a quasi-shear wave is orthogonal to its propagation direction and lies in plane (YZ plane).

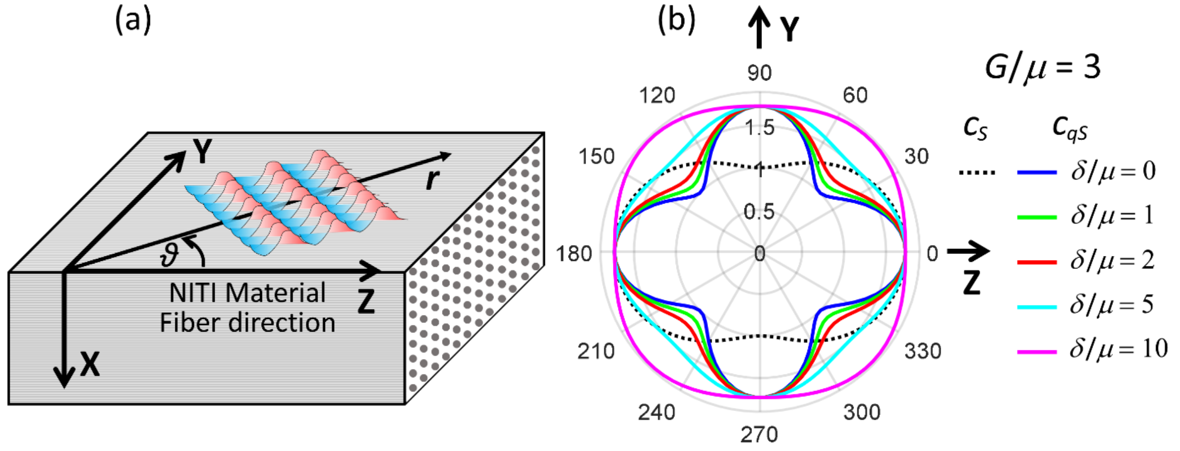

Supplementary Figure S2*:* **(a)** – Diagram of wave propagation in a fast-axis NITI medium. Fiber direction is in the Z direction and the wave propagation direction is represented by $\boldsymbol{r}$ in the YZ plane. **(b)** – Phase velocity of bulk shear, c_S, (black dashed line) and quasi-shear, c_{qS}, waves in YZ plane of fast-axis NITI medium for $G/\mu = 3$ and $\delta/\mu = 0, 1, 2, 5, 10$ as a function of angle with respect to the fiber direction **z**. Speeds are scaled to the speed of a shear wave ($\sqrt{\mu/\rho}$) along the Y direction.

Propagation speeds for shear and quasi-shear waves in the YZ plane are illustrated in Suppl. Fig. S2b scaled to the shear wave speed ($\sqrt{\mu/\rho}$) along the Y direction for $G/\mu = 3$ and variable δ/μ. Clearly, the shear wave speed c_S (black dotted line) monotonically decreases from $\sqrt{G/\rho}$ along the Z direction (e.g., along the fibers in skin) to $\sqrt{\mu/\rho}$ along the Y direction (e.g., across the fibers).

Propagation of the quasi-shear wave (solid lines) is more complicated. Along the fibers, it is converted to a pure shear wave with a speed of $\sqrt{G/\rho}$. It is also converted to a pure shear wave propagating across the fibers (at $\vartheta = 90°$). Because this wave is polarized in the YZ plane, the propagation across fibers corresponds to its polarization along fibers and vice versa. Both situations result in the same propagation speed $\sqrt{G/\rho}$. It is interesting that at $\vartheta = 45°$, the propagation speed does not depend on G and approaches $\sqrt{\mu/\rho}$ as $\delta \to 0$.

Supplementary Note 3: Rayleigh wave Propagation in a linear elastic, fast-axis NITI material

A well-defined surface imposes additional constraints on propagating waves so that traction forces should be taken into account. A real root of the wave motion equation corresponds to a Rayleigh wave with amplitude decreasing exponentially with depth. In nearly incompressible isotropic materials, its speed is directly related to c_S, $c_R \approx 0.9553 c_S$. Propagation velocities of shear and Rayleigh waves can then be converted into the Young's modulus $E = 3\rho c_S^2$.

Now consider wave propagation in the same YZ plane, but over the surface of a fast-axis NITI medium. In addition to the unit propagation vector $\mathbf{m}$ = [0, sin ϑ, cos ϑ], a surface unit normal vector $\mathbf{n} = [1, 0, 0]$ should be introduced. The solution for the Rayleigh wave speed c_R can be found using the Stroh formalism with 3x3 matrices [6]:

$$Q_{ik} = c_{ijkl} m_j m_l - \rho c^2 \delta_{ik} , \tag{S16}$$

$$R_{ik} = c_{ijkl} m_j n_l , \tag{S17}$$

$$T_{ik} = c_{ijkl} n_j n_l . \tag{S18}$$

Matrices (S16) – (S18) can be combined to form the 6x6 Stroh eigenvalue problem:

$$\mathbf{N} \begin{bmatrix} \mathbf{a} \\ \mathbf{b} \end{bmatrix} = p \begin{bmatrix} \mathbf{a} \\ \mathbf{b} \end{bmatrix} , \tag{S19}$$

where k is the wave number and the matrix **N**

$$\mathbf{N} = \begin{bmatrix} -\mathbf{T}^{-1}\mathbf{R}^{\mathrm{T}} & \mathbf{T}^{-1} \\ \mathbf{R}\mathbf{T}^{-1}\mathbf{R}^{\mathrm{T}} - \mathbf{Q} & -\mathbf{T}^{-1}\mathbf{R}^{\mathrm{T}} \end{bmatrix} . \tag{S20}$$

Eigenvectors contain polarization $\mathbf{a}$ and traction $\mathbf{b}$ vectors of harmonic waves that satisfy the free surface boundary conditions and whose displacement $\mathbf{u}$ and traction $\mathbf{t}$ are represented in the form:

$$\mathbf{u} = \mathbf{a} \boldsymbol{e}^{ik(\mathbf{m}\cdot\mathbf{r} + p\mathbf{n}\cdot\mathbf{r} - ct)} , \tag{S21}$$

$$\mathbf{t} = ik\mathbf{b}\boldsymbol{e}^{i(k\mathbf{m}\cdot\mathbf{r} - ct)} . \tag{S22}$$

The amplitude of a Rayleigh wave must decay with depth, and therefore p has a positive imaginary part. Six eigenvalues occur in complex conjugate pairs $\begin{bmatrix} \mathbf{a} \\ \mathbf{b} \end{bmatrix}$, but only three depth-decaying modes form Rayleigh waves. In addition, the free surface boundary condition implies that the linear combination of tractions must equal zero:

$$\sum_{t=1}^{3} c_i \mathbf{t}_i = 0 \quad , \tag{S23}$$

which can be rewritten as:

$$ik\begin{bmatrix} | & | & | \\ \mathbf{b}_1 & \mathbf{b}_2 & \mathbf{b}_3 \\ | & | & | \end{bmatrix}\begin{bmatrix} c_1 \\ c_2 \\ c_3 \end{bmatrix} e^{ik(\mathbf{m}\cdot\mathbf{r}-ct)} = \mathbf{Bc} = \mathbf{0}\,, \tag{S24}$$

with a nontrivial solution if and only if the determinant of $\mathbf{B}$ is zero.

To solve for the Rayleigh wave speed, an iterative algorithm is used. At each iteration, a trial wave speed v is considered, and the Stroh eigenvalue problem is solved to obtain the relevant eigenvectors, as described above. The traction vectors are extracted and used to form the matrix $\mathbf{B}$ and calculate its determinant. This process is repeated until the absolute value of the determinant is minimized. The resulting wave speed corresponds to the Rayleigh wave speed c_R.

The simplest scenario occurs when a Rayleigh wave propagates across fibers in the fast-axis NITI medium. Its speed has the same relationship with the shear wave speed as in an isotropic material, and it is fully defined by modulus μ without any dependence on G and δ.

Obtaining an analytic solution for the Rayleigh wave speed at an arbitrary angle to the symmetry axis Z (fiber direction for skin) and its dependence on G/μ and δ/μ is complicated and possible only for a few cases. It can be shown (see Ref [7]) that that the velocity of Rayleigh waves along fibers obeys the secular equation below, similar to that for the isotropic case [8], but with different coefficients:

$$\xi^3 - 2\kappa\xi^2 + \kappa(2G_1 + \kappa)\xi - G_1\kappa^2 = 0\,, \tag{S25}$$

where

$$\begin{aligned} \xi &= \rho C_R^2/\mu, \\ \kappa &= (4\mu + \delta)/\mu \\ G_1 &= G/\mu. \end{aligned} \tag{S26}$$

Equation (S25) can also be written in an alternative form:

$$\xi^3 - 8(1+\tfrac{\delta}{4\mu})\xi^2 + 24\left(\tfrac{2}{3}\left(1+\tfrac{\delta}{4\mu}\right)\left(1+\tfrac{\delta}{4\mu}+\tfrac{G}{2\mu}\right)\right)\xi - 16\tfrac{G}{\mu}(1+\tfrac{\delta}{4\mu})^2 = 0\,, \tag{S27}$$

As noted in Ref [5], the speed of the quasi-shear wave is not limited by $\sqrt{G/\rho}$ and can change broadly with δ; however, the speed of a Rayleigh wave in a NITI medium cannot exceed $\sqrt{G/\rho}$. This limit is reached when $\delta/\mu \to \infty$.

Note that in the isotropic limit, the equation reduces to the well-known Rayleigh equation [7]:

$$\xi^3 - 8\xi^2 + 24\xi - 16 = 0\,. \tag{S28}$$

Supplementary Figure S3 shows the behavior of the Rayleigh wave speed in the fast-axis NITI medium (typical for skin and muscles) at different values of δ/μ. Clearly, the absolute limitation

on the Rayleigh wave speed is the speed c_S of bulk shear waves polarized perpendicular to the surface (along the X-axis).

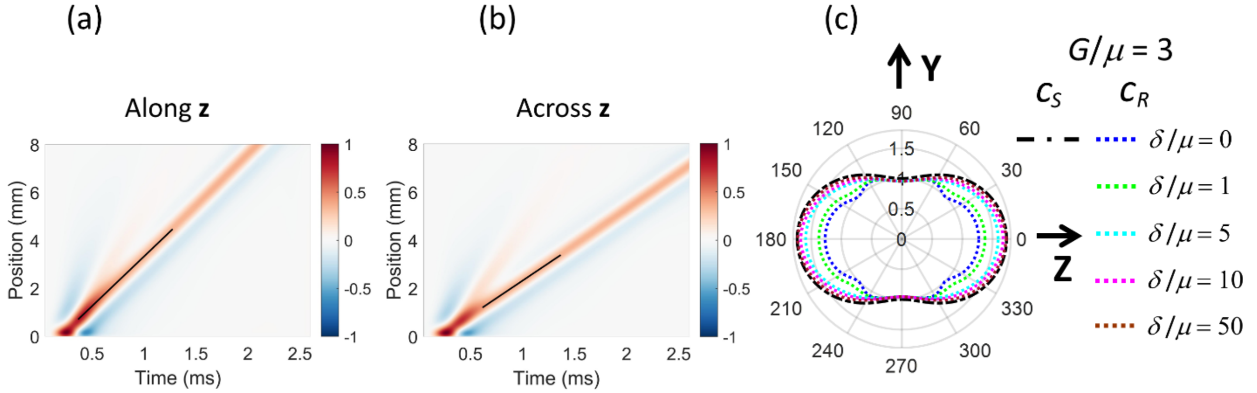

Supplementary Figure S3*:* Rayleigh mechanical waves on the surface of a bulk NITI medium. Simulated in OnScale wavefields for propagation along **(a)**, and across **(b)** the symmetry axis **z** (fiber direction) of a NITI medium for $G/\mu = 3$ and $\delta/\mu = 1$. Black solid lines in panels **(a)** and **(b)** correspond to calculated Rayleigh wave speed. **(c)** Speed, c_R, of propagating Rayleigh wave in YZ plane of a fast-axis NITI medium scaled to the shear wave speed ($\sqrt{\mu/\rho}$) along the Y direction for $G/\mu = 3$ and $\delta/\mu = 0, 1, 5, 10, 50$ as a function of angle with respect to the symmetry axis. The speed c_S of bulk shear waves polarized perpendicular to the surface is shown by the black dash-dot line for comparison.

Supplementary Note 4: Influence of a thin isotropic layer (epidermis) on Rayleigh wave propagation in a fast-axis NITI material

Skin is a multilayered structure consisting of epidermis, dermis, and hypodermis layers. Here we investigate skin's elastic properties assuming that they are dominated by the dermis and that the dermis represents a NITI medium. The epidermis in skin is much thinner than the dermis and, therefore, cannot strongly affect the skin's elastic behavior and its deformation. However, adding a layer on top of a NITI material might affect the propagation speed of a Rayleigh wave depending on the relationship between its wavelength and the layer thickness, i.e. on the parameter kh (h is the layer thickness and k is 2π times the wavelength). Indeed, when the layer thickness is much smaller than the wavelength, $kh \ll 1$, the speed of a Rayleigh wave is defined by the speed in the substrate, i.e., the layer can be ignored [8]. In contrast for a very thick layer, $kh \gg 1$, the Rayleigh wave speed is solely determined by the properties of the layer [8, 10].

In our AμT-OCE measurements in skin, the maximum frequency of Rayleigh waves is usually limited to about 3 kHz, which corresponds to about 1 mm wavelength in dermis. It is evident that the epidermal thickness around 50 μm to 100 μm is much smaller that the wavelength. Thus, intuitively, the epidermis should not affect the Rayleigh wave speed, but considering that the difference in Young's moduli between epidermal and dermal layers can be dramatic, it is worth checking this effect quantitatively.

The influence of medium loading with a thin layer on the propagation characteristics of Rayleigh waves was investigated in multiple past studies, mostly for solids. In the majority of cases, a softer coating was considered [8]. Studies for a stiffer coating than the substrate are limited [10]. However, Rayleigh waves in a layered soft media, to our knowledge, were not quantitatively described.

For skin the substrate (dermis) is an anisotropic NITI medium, whereas the epidermis was shown to be isotropic (at least optically) in multiple studies (see Ref [11], for example). Considering this structure analytically is not a simple task; therefore, numerical simulations were performed in OnScale instead.

We have used OnScale to build numerical AμT-OCE models in several numerical studies before, including simulating nearly incompressible anisotropic media [4]. Details on the simulation parameters for nearly incompressible media and numerical model testing can be found in Refs [4, 11]. The simulation script for the model used here is shared in Supplementary Software Library. Supplementary Figures S4a,b present diagrams of wave propagation along and across fibers. Examples of Rayleigh wavefields, and corresponding 2-D spectra, are shown in Supplementary Figures S4c-f.

Because epidermis provides mainly protection, we set its shear modulus to be $\mu = 500$ kPa, which is much larger than that in dermis. Note that the Young's modulus in epidermis may be different in different body sites, may vary with age, gender, and an individual's lifestyle and environment, and many other conditions. As seen in Suppl. Figure S4c-f, even if the modulus in

the epidermis is 50 time larger than that for dermis, it does not change the Rayleigh wave speed appreciably. Thus, we can conclude that ignoring the epidermis in reconstructing elastic moduli in skin is justified for the case of $kh \ll 1$.

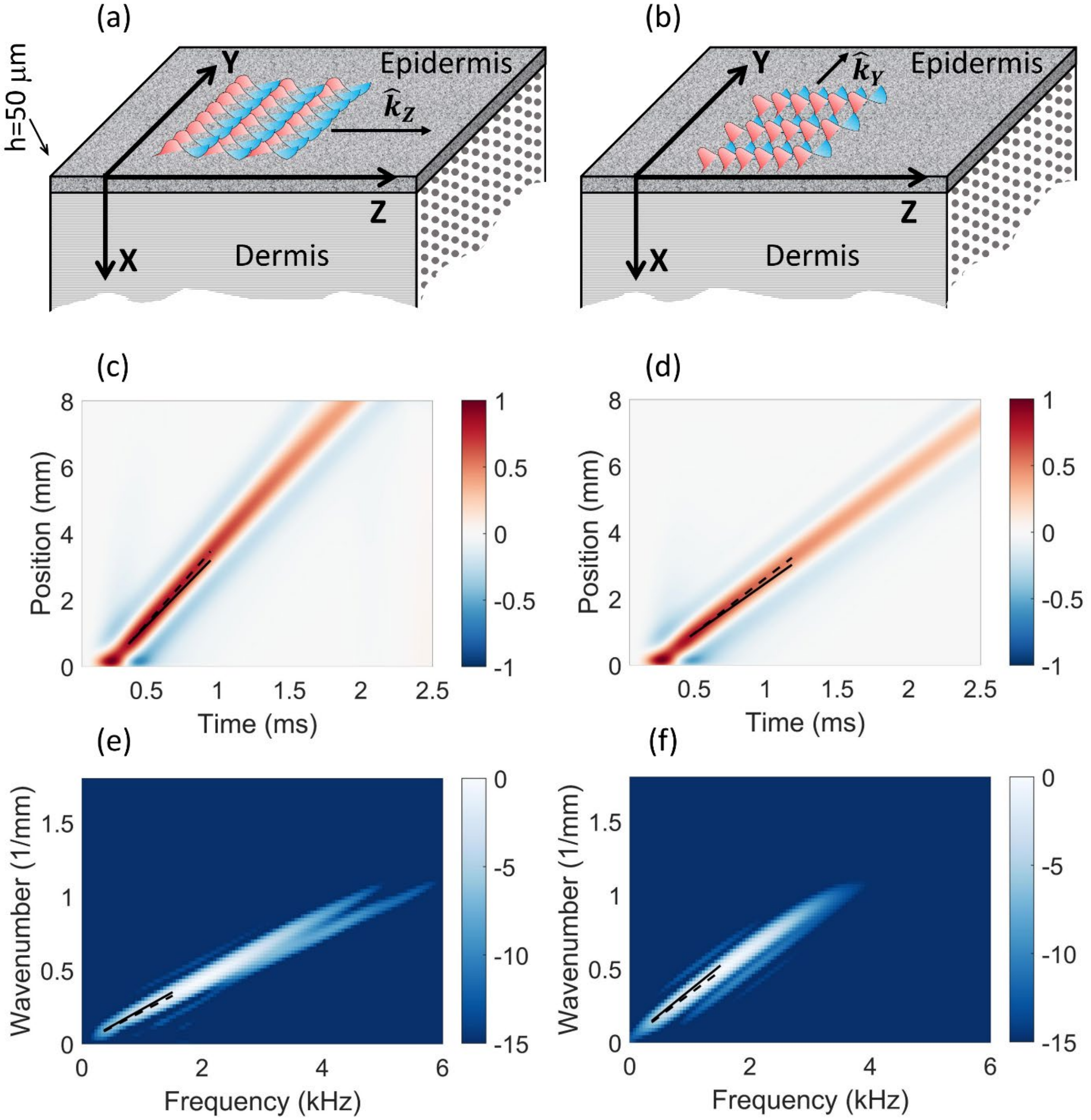

Supplementary Figure S4: Diagram of Rayleigh wave propagation over the surface of a 2-layer (epidermis/dermis) system along **(a)** and across **(b)** the symmetry axis of the second layer. Epidermis is modeled as a 50 μm thick isotropic material with $\mu = 500$ kPa; dermis is modeled as a fast-axis NITI hemispace with $\mu = \delta = 10$ kPa, $G = 30$ kPa. Simulated wavefields of vertical vibration velocity and 2D Fourier spectra of Rayleigh waves are shown accordingly for propagation along **(c)**, **(e)** and across **(d)**, **(f)** the symmetry axis of the NITI medium. Solid lines in panels **(c)**-**(f)** correspond to Rayleigh wave speeds in a bulk NITI medium, and dashed lines correspond to fitting. The color bar is linear in panels **(c)** and **(d)** and shown in arbitrary units. The color bar for spectral amplitudes in panels **(e)** and **(f)** is shown in dB relative to the maximum signal.

Supplementary Note 5: Guided waves in a bounded (with hypodermis) NITI layer (epidermis).

As demonstrated in Supplementary Note 4 above, a thin epidermal layer has little influence on the propagation of Rayleigh waves in dermis due to its small thickness compared to the wavelength, i.e. under the condition $kh \ll 1$. However, the thickness of dermis itself is not infinite and usually on the order of 1 mm, representing a $kh \approx 1$. For this case, guided wave behavior can be expected. Below, we investigate how a mismatch between elastic properties of dermis and hypodermis influence mechanical wave propagation over the skin surface.

Because the hypodermis (or subcutaneous tissue) is mainly connective tissue with a large fraction of fat and almost no fibers, we assume subcutaneous tissue to be isotropic and having a lower Young's modulus than that in dermis due to the very low elastic modulus of fat. In simulations, we considered it to be $E_{sc} = 2$ kPa. The hypodermis was simulated as a hemispace bounded with the dermis on top (see Suppl. Figure S5a,b).

Since the dermal layer is anisotropic (assumed as a fast-axis NITI medium), we explored wave propagation along and across fibers. The epidermis was assumed to be 50 μm thick and the dermis was assumed to be 1 mm thick. The elastic properties of epidermis and dermis are listed in the caption to Suppl. Figure S5 and are the same as those used in Suppl. Figure S4.

As seen in Suppl. Figure S5c,d, bounding of the dermal layer on its bottom modifies the wavefields for Rayleigh wave propagation both along and across the symmetry axis (fiber direction). The wavefield also changes for wave propagation along other directions in the YZ plane. Fourier analysis shows evidence of guided waves when propagation over multiple wavelengths is used for analysis. However, at small distances from the AμT source, as shown by the fit lines in Figure S5c,d, the wavefields closely resemble the unbounded case, strongly suggesting that group velocity measurements represent Rayleigh wave speeds.

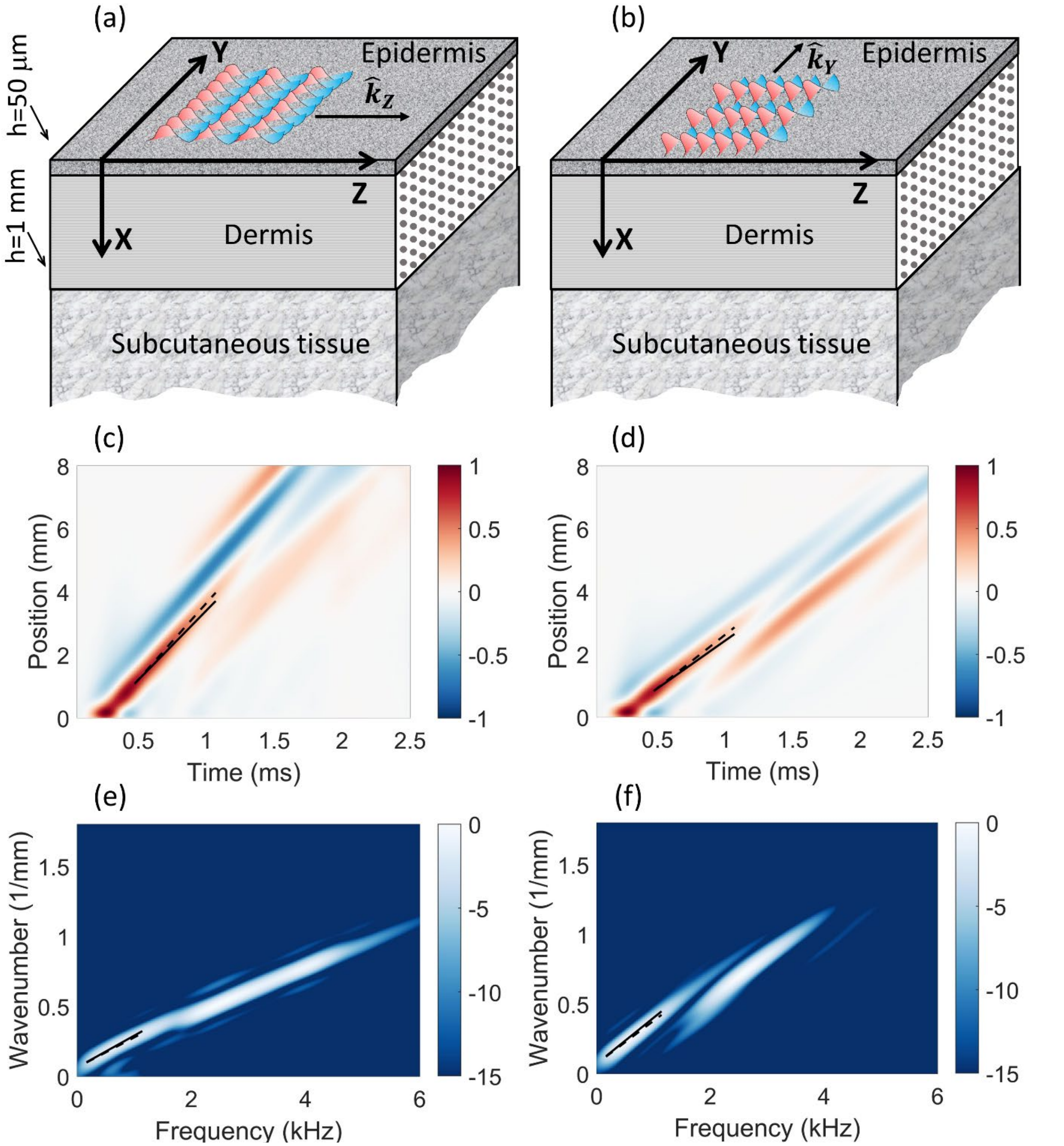

Supplementary Figure S5: Diagram of Rayleigh wave propagation over the surface of a 3-layer (epidermis/dermis/subcutaneous tissue) system along **(a)** and across **(b)** the symmetry axis of the second layer. Epidermis is modeled as a 50 μm thick isotropic material with $\mu = 500$ kPa; dermis is modeled as a 1 mm thick fast-axis NITI medium with $\mu = \delta = 10$ kPa, $G = 30$ kPa; and subcutaneous tissue is modeled as an isotropic hemispace with $\mu = 2$ kPa. Simulated OnScale wavefields and 2D Fourier spectra of Rayleigh waves are shown accordingly for propagation along (c), (e) and across (d), (f) the symmetry axis of the NITI medium. Solid lines in panels **(c)**-**(f)** correspond to Rayleigh wave speeds in a bulk NITI medium, and dashed lines correspond to fitting. The color bar is linear in panels **(c)** and **(d)** and shown in arbitrary units. The color bar for spectral amplitudes in panels **(e)** and **(f)** is shown in dB relative to the maximum signal.

Supplementary Note 6: Experimental study of guided waves in ex vivo chicken skin.

AμT-OCE experiments were performed in ex vivo chicken samples to investigate the influence of subcutaneous tissue on the propagation of mechanical waves in skin. Chicken drumsticks were bought in a grocery store.

In the first set of experiments, whole chicken drumsticks (Suppl. Fig. S6a) were explored. Waveforms were recorded at different propagation directions for several positions in the drumstick. Ten different waveforms of surface-propagating mechanical waves were recorded. A typical wavefield for the whole drumstick is represented in Suppl. Fig. S6d. Clearly, guided behavior is not seen in this case, and the wavefield can be fit with a linear function, indicating nondispersive wave propagation. The slope of the wavefield corresponds to the group velocity of the propagating wave, which equals the Rayleigh wave speed when dispersion is not observed. The mean propagating wave speed calculated by the ensemble of 10 measurements was $c_R = (7 \pm 1)$ m/s corresponding to the shear modulus $\mu = (54 \pm 15)$ kPa, assuming chicken skin isotropy. The calculated standard deviation corresponds to the statistical variation of the propagation wave speed that can be due to sample inhomogeneity or some anisotropy.

In the second set of experiments, skin was removed from muscle (see Suppl. Fig. S6b) and placed on top of a container filled with water (see Suppl. Fig. S6c). In this case, skin had an interface with a medium having zero shear modulus, which should produce guided waves in skin with (see Suppl. Fig. S6e) a strong geometrical dispersion of wave velocity (see Suppl. Fig. S6f) typical for Lamb/Scholte waves [8]. Fitting the dispersion curve with the analytical expression for a Scholte wave propagating in the layer bounded with air on one side and with water on the other [12, 13], gives a modulus estimate $\mu = (53 \pm 2)$ kPa. Because estimation of the mean is performed by fitting over a wide frequency range, its accuracy is much higher than that obtained from the mean group velocity.

The results of this study strongly support the assumption that when skin is bounded with a tissue having a similar shear modulus, guided behavior is not observed. Hence, wave propagation over the skin surface may be considered as a Rayleigh wave propagating over the surface of a bulk material with moduli corresponding to that of skin. A detailed investigation of the effect of subcutaneous tissue on wave propagation in skin is outside of the scope of this study. Definitely, when skin is thin and located on top of bones, or in close proximity to blood vessels, guided propagation is much more possible.

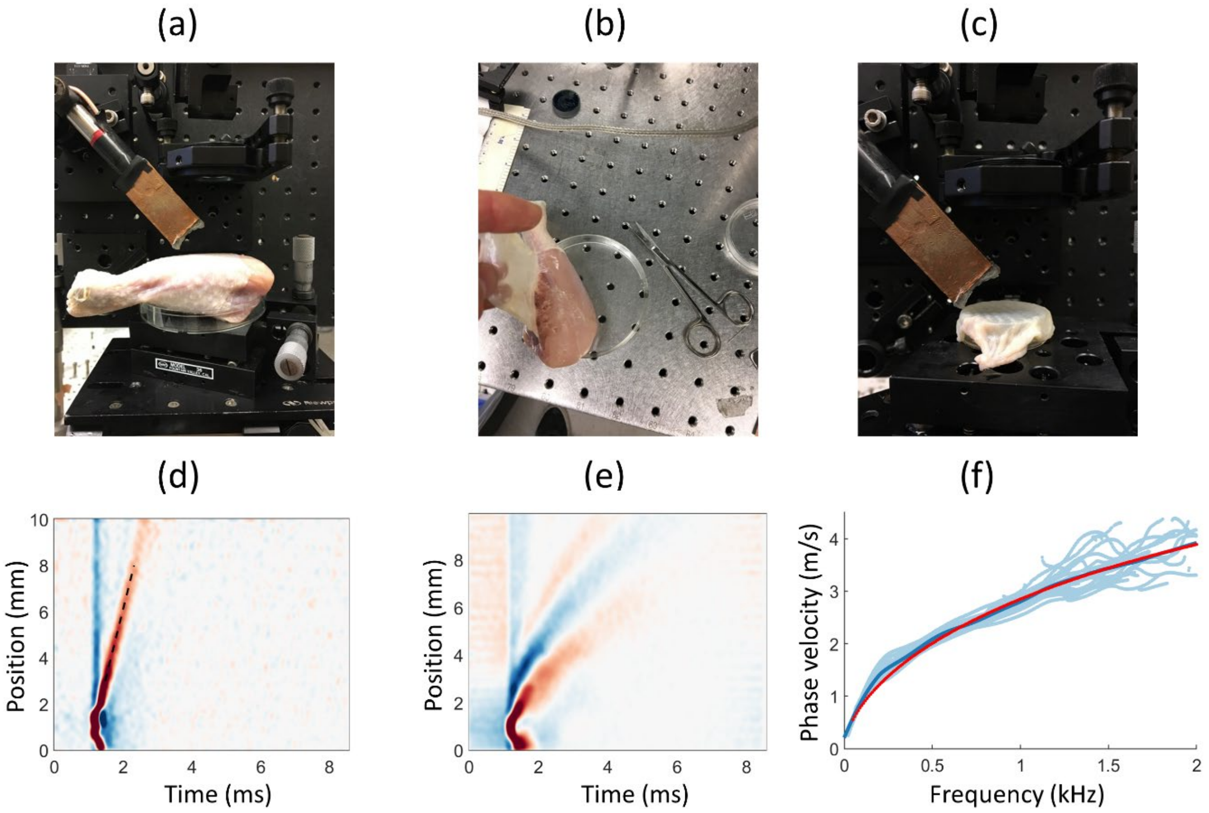

Supplementary Fig. S6. **Propagation of AμT-generated mechanical waves in chicken skin. (a)** - Photograph for experiment where the mechanical wave propagates over the surface of whole chicken drumstick. **(b)** - Photograph of the skin removed from muscle. **(c)** - Photograph of chicken skin positioned on top of a container filled with water after being removed from muscle. **(d)** - Typical wavefield for the case **(a)**. **(e)** - Typical wavefield for the case **(c)**. Dispersion of wave velocity for case **(c)**. Bold blue line corresponds to the averaged dispersion curve; red line corresponds to its fit to an analytical solution [12, 13].

Supplementary References

1. Lai, W.M., Rubin, D., Krempl, E. *Introduction to continuum mechanics*. 4th edition. (Butterworth-Heinemann, 2009).
2. Auld, B. A. *Acoustic fields and waves in solids*. (Krieger Pub Co, 1990).
3. O'Donnell, M., Skovoroda, A.R. Prospects for elasticity reconstruction in the heart. *IEEE Tran. UFFC* **51**(3), 322-328 (2004).
4. Pitre, J. J. *et al.* Nearly-incompressible transverse isotropy (NITI) of cornea elasticity: model and experiments with acoustic micro-tapping OCE. *Sci. Rep.* **10,** 12983 (2020).
5. Rouze, N.C. *et al.* Finite element modeling of impulsive excitation and shear wave propagation in an incompressible, transversely isotropic medium. *J. Biomech*. **46**(16), 2761-2768 (2013).
6. Tanuma, K. Stroh Formalism and Rayleigh Waves. *J. Elasticity* **89**, 5–154 (2007).
7. Destrade, M. Surface waves in orthotropic incompressible materials. *JASA* **110**(2), 837-840 (2001).
8. Viktorov, I. A. *Rayleigh and Lamb waves: physical theory and applications*. (Plenum press, 1970).
9. Carroll, R.G. *Elsevier's Integrated Physiology*. (Elsevier, 2007).
10. Shuvalov, A. L, Every, A. G. Characteristic features of the velocity dispersion of surface acoustic waves in anisotropic coated solids. Ultrasonics. **40**(1-8), 939-42 (2002).
11. Pircher, M. *et al*. Three dimensional polarization sensitive OCT of human skin in vivo. Opt. Express **12**(14): 3236-3244 (2004).
12. Pitre, J. J. *et al.* Super-shear evanescent waves for non-contact elastography of soft tissues. *Appl. Phys. Lett.* **115**, 083701 (2019).
13. Kirby, M.A. *et al.* Optical coherence elastography in ophthalmology. *J. Biomed. Opt.* **22**(12), 1-28 (2017).
